# Papuan Admixture Predated the Settlement of Palau

**DOI:** 10.1016/j.cell.2026.02.011

**Published:** 2026-03-10

**Authors:** Yue-Chen Liu, Joanne Eakin, Jolie Liston, Rosalind Hunter-Anderson, Calvin Emesiochel, Kiblas Soaladaob, Sunny O. Ngirmang, Olivia Cheronet, Carla S. Hadden, Alexander Cherkinsky, Matthew Spriggs, Keith Prufer, Swapan Mallick, Nadin Rohland, Ron Pinhasi, David Reich

**Affiliations:** 1 Key Laboratory of Vertebrate Evolution and Human Origins, Institute of Vertebrate Paleontology and Paleoanthropology, Chinese Academy of Sciences, Beijing, China; 2 Department of Human Evolutionary Biology, Harvard University, Cambridge, MA, USA; 3 Department of Genetics, Harvard Medical School, Boston, MA, USA; 4 Independent Researcher, Albuquerque, NM, USA; 5 Palau and Micronesian Heritage Consulting, LLC., Koror, Palau; 6 Independent Researcher, Albuquerque, NM, USA; 7 Palau Bureau of Cultural and Historical Preservation, Koror, Republic of Palau; 8 Department of Evolutionary Anthropology, University of Vienna, Vienna, Austria; 9 Human Evolution and Archaeological Sciences, University of Vienna, Vienna, Austria; 10 Center for Applied Isotope Studies, Athens, GA, USA; 11 Vanuatu National Museum, Vanuatu Culture Centre, Port Vila, Vanuatu; 12 School of Archaeology and Anthropology, Australian National University, Canberra, Australia; 13 Department Anthropology, University of New Mexico, Albuquerque, NM, USA; 14 Center for Stable Isotopes, University of New Mexico, Albuquerque, NM, USA.; 15 Broad Institute of MIT and Harvard, Cambridge, MA, USA; 16 Howard Hughes Medical Institute, Harvard Medical School, Boston, MA, USA; 17 Lead contact

**Keywords:** Palau, First Remote Oceanians, Micronesia, Austronesian, Ancient DNA

## Abstract

The first people reached Remote Oceania before 3000 years ago (BP), arriving roughly simultaneously in the southwest Pacific, the Marianas Archipelago, and Palau. However, no genome-wide ancient DNA data have been available from Palau, a gap we address by reporting 21 individuals from four archaeological sites dating between 2900 and 500 BP. All had approximately 60% ancestry related to East Asians and 40% to Papuans, similar to present-day Palauans, the longest stretch of population continuity anywhere in Remote Oceania. The lengths of contiguous Papuan ancestry segments in the oldest individuals show that major admixture between Papuans and East Asians in the ancestors of all sampled Palauans began prior to first settlement. This differs from the pattern in the southwest Pacific, where sampled individuals of the Lapita archaeological culture from three different islands had almost entirely East Asian ancestry, with large amounts of Papuan admixture observed only hundreds of years later.

## IN BRIEF

Ancient DNA data from Palau reveals long-standing genetic continuity, and shows that the major admixture between Papuans and East Asians that occurs in many Remote Oceanians today, in some cases was present in first settlers more than 3200 years ago.

## Graphical Abstract

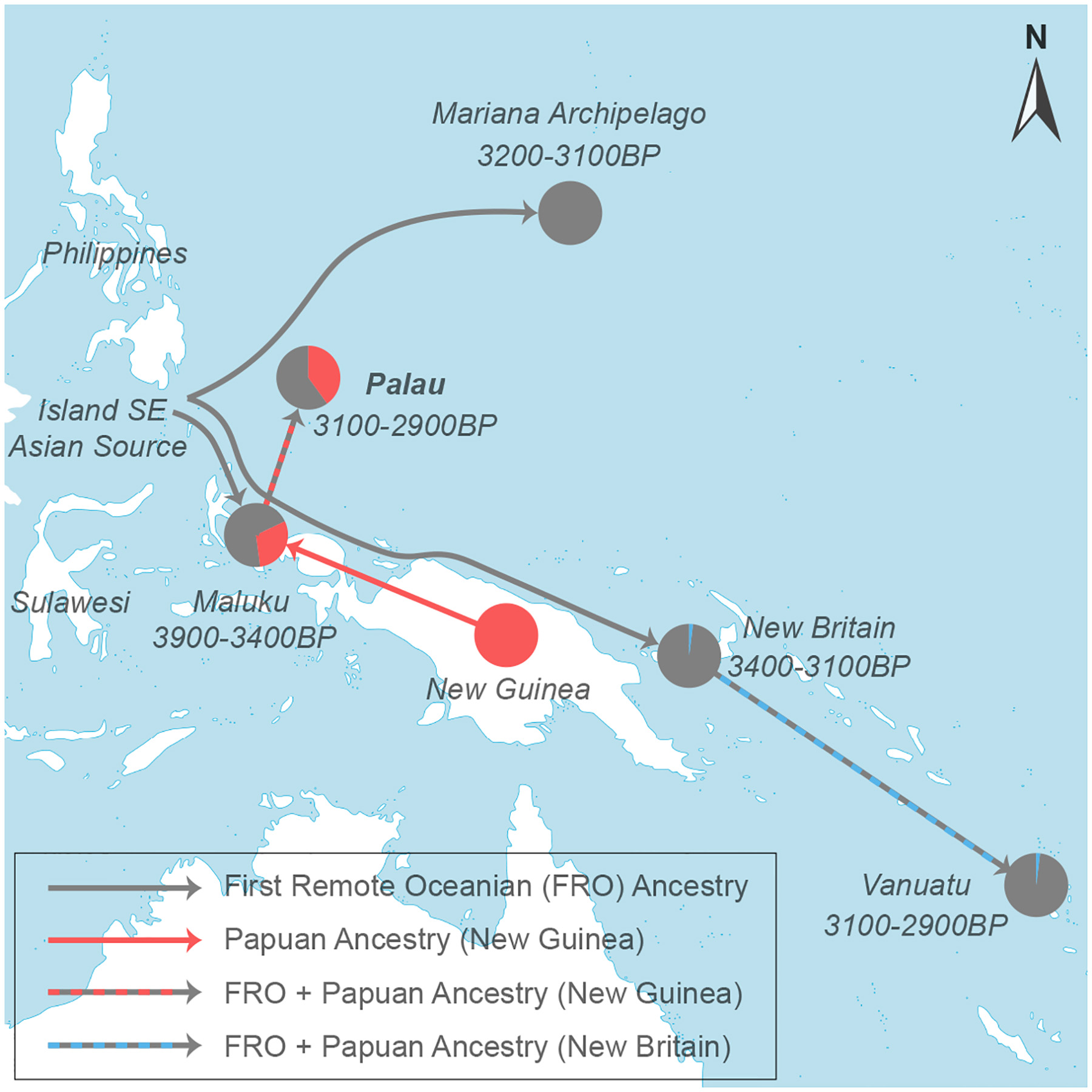

## INTRODUCTION

Ancient DNA data obtained from people of the Lapita Archaeological Complex show that all analyzed individuals from this first culture of the southwest Pacific, spanning 3000–2500 BP and sampled from three sites on two different archipelagos, have on the order of 96–100% East Asian ancestry^1–9^, derived from a population that no longer exists in unmixed form and has been called “First Remote Oceanian” (FRO_SouthwestPacific_)^2,3,5,6^. Genetic analysis indicates the East Asian ancestors of these individuals were most closely related to the ancestors of present-day Cordillerans (e.g., Kankanaey) from northern Luzon in the Philippines^1–8^, and more distantly related to Neolithic and Iron Age people in Taiwan^10,11^. Beginning with post-Lapita individuals dating to around 2500 BP, there was a large-scale, primarily male-driven migration into the southwest Pacific of almost entirely Papuan ancestry from the lineage that was predominant in New Guinea and adjacent islands for tens of thousands of years^12,13^, and is specifically related to people of New Britain in the Bismarck Archipelago (Papuan_NewBritain_)^3–6^. Today, most Indigenous Ni-Vanuatu people possess over 80% Papuan ancestry^3^, which also spread east into Fiji, and further east into Polynesia, where today at least 25% of the ancestry is Papuan^2^.

In Micronesia, the earliest ancient DNA data come from Unai Period individuals from the southern Marianas (Guam) dated to 2800–2400 BP^6,8^. Their ancestry (FRO_Marianas_) is a close sister group to that of Lapita culture individuals of the Southwest Pacific (FROSouthwestPacific). Individuals from the Latte Period in the Marianas (Guam and Saipan) derived 15% ancestry from another East Asian source related to the primary East Asian ancestry (FRO_Palau_) of the present-day people in Palau, which mixed with FRO_Marianas_ around 2400–1700 BP^6^, plausibly during the Marianas Huyong Period, marked by a new pottery type and inland settlement expansion^14^. Central Micronesian islands such as Pohnpei and Chuuk were occupied later, and shared with people of the Marianas and the Southwest Pacific evidence of admixture occurred 2500–2000 BP^6^. But in central Micronesia, the mixing sources were different, with male Papuan_NewGuinea_ (rather than Papuan_NewBritain_ as in the southwest Pacific), and female FRO_SouthwestPacific_ ancestry (differing from East Asian sources of FRO_Marianas_ and FRO_Palau_ elsewhere in western Micronesia)^6^.

The history of Palau is central to the story of human advent in Remote Oceania. Humans first occupied Palau’s limestone Rock Islands and volcanic Babeldaob about 3200 BP (Table S1), around the same time as the Marianas and Southwest Pacific were reached in separate migration streams. One reconstruction assigns the Palauan language to be part of a residual subgroup within the Malayo-Polynesian (MP) language family, which also comprises languages spoken in the Philippines, the Marianas, western Indonesia (as far as Sulawesi and Borneo), and mainland Southeast Asia^15^. Another reconstructions assigns Palauan, CHamoru/Chamorro in the Marianas (“CHamoru” is the expressed preference of Guam language specialists, and “Chamorro” is used elsewhere in the Marianas^6^), and the Oceanic Austronesian languages (spoken across much of Remote Oceania) to equally distinct branches^16,17^. These reconstructions agree in suggesting that the languages spoken in Palau, the Marianas, the Southwest Pacific, and other parts of Remote Oceania share a common origin: a single ancestral Remote Oceanian population that later split into at least three distinct branches. Genetic clues also point to a critical role for Palau^6^. Palau harbors the first branching of the three FRO lineages (FRO_Palau_), contributing to about 60% of the ancestry of modern Palauans, the rest being primarily male-derived Papuan ancestry related to that of mainland New Guinea (Papuan_NewGuinea_), with the admixture date estimated at 2800–2000 BP. Genetic evidence from present-day Palauans, along with ancient DNA from Central Micronesia, the Marianas, and the Southwest Pacific, reveals four distinct instances of major sex-biased migration and interaction in Remote Oceania between 2500 and 2000 BP. Without DNA from ancient Palauans, the role of this southwesternmost archipelago in Micronesia, has remained unclear.

## RESULTS

**Figure 1.**
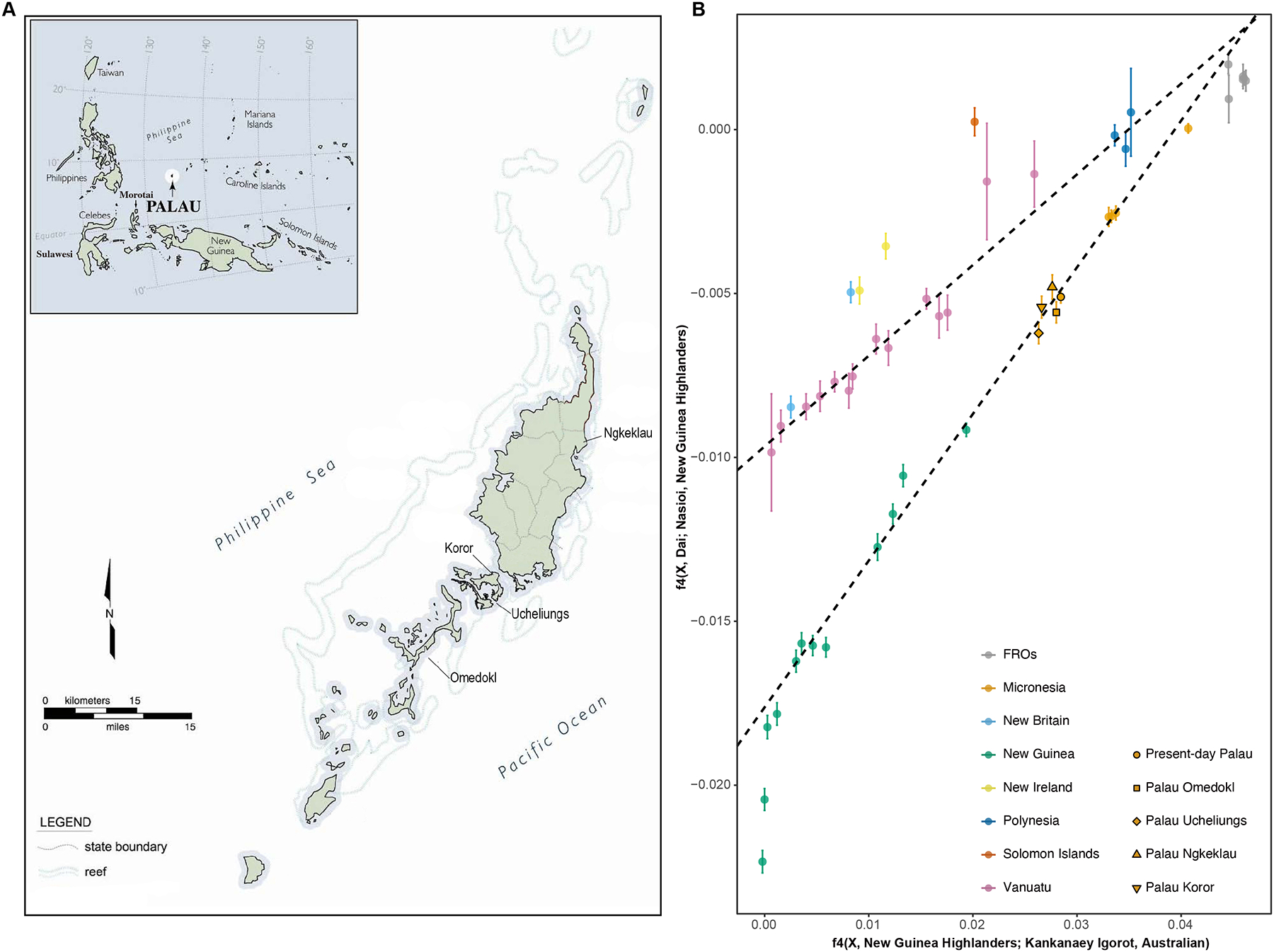
Sample locations, genetic ancestry, and admixture time. A) Palau specimen collection locations. Koror specimens are from the Ngermereues Ridge burial cave at Ngesaol on the island of Koror. Omedokl and Ucheliungs are Rock Islands. The Ngkeklau specimens are from an unknown location due to poor documentation in the physical anthropology collection, but are likely from the Ngkeklau area of northeastern Babeldaob. B) Papuan affinities of diverse Pacific Islanders. We show one standard error in each direction on the y-axis. We merged Lapita individuals from Vanuatu and Tonga.

We analyzed 37 skeletal specimens from four archaeological sites in Palau (Figure 1A): 22 from the Ngermereues Ridge burial cave (Site OR-3:30) in Ngesaol on Koror (“Koror Quarry”); six from the Ucheliungs Rock Island burial cave (Site OR-14:8) (“Ucheliungs”); four from the Omedokl Rock Island burial cave (Site OR-15:8) (“Omedokl”), and five possibly from the Ngkeklau area in northeastern Babeldaob (“Ngkeklau”) (Tables S2.1 and S2.2). We generated genome-wide ancient DNA data using in-solution enrichment for more than a million single nucleotide polymorphisms (SNPs). Including SNPs near the targeted ones, we analyzed data from up to about 2 million polymorphic SNPs. After filtering to unique individuals with no evidence of contamination (Method Details), we retrieved data for 21 individuals: 12 from Koror Quarry, 3 from Ucheliungs, 3 from Omedokl, and 3 from Ngkeklau (Table 1). Among these individuals, we did not find close relatives, long runs of homozygosity (ROH) (Table S2.3), or substantial sharing of identity-by-descent (IBD) segments (Table S2.4).

Establishing an accurate chronology is crucial for the key findings of this study. We generated 17 accelerator mass spectrometry (AMS) radiocarbon dates from 13 individuals for which we also obtained genome-wide ancient DNA data. For each of these dates, we also obtained measurements of carbon and nitrogen isotopes providing information about the preservation of the collagen (Table S3.1). Twelve samples were prepared and dated at the Center for Applied Isotope Studies, University of Georgia (UGAMS). We prepared an additional five at the Center for Stable Isotopes (CSI) at the University of New Mexico, followed by dating at Pennsylvania State University’s Radiocarbon Laboratory (PSUAMS). We rejected dates that did not have a carbon to nitrogen (C:N) atomic weight ratio in the recommended range of 2.9–3.4. When we obtained two dates for a sample – one based on bone collagen pretreated by ultrafiltration or amino acid isolation to remove possible contaminants, and one without such pretreatment – we always used the date based on pretreatment. After applying these procedures, we had direct dates on 11 unique samples. Two of the dates from Ucheliungs were close to the time of settlement of Palau and within the range of expected radiocarbon dates on cultural material at the site (Figure S1 and Table S3.2): I41048 at 2924–2710 calBP (date from ultrafiltration), and I41049 at 2776–2495 calBP (date from amino acid purification). A dietary correction for a radiocarbon date on bone collagen must reflect the proportion of marine carbon incorporated into collagen routed from dietary protein, not the proportion of marine foods in the total diet. For our main analyses, we calibrated the dates using an assumption of 50% marine and 50% terrestrial protein^18^. We also estimated dates with a range of other assumptions about marine proportion in the protein and marine reservoir offset (Tables S3.1 and S3.3) and found that our key finding of major admixture between Papuans and East Asians predating the initial settlement of Palau was robust.

### At least 40% Papuan ancestry in Palau for almost three thousand years

To obtain an overview of population structure, we carried out principal components analysis (PCA) on approximately three million SNPs by integrating data from diverse Pacific populations genotyped on different arrays (Method Details) (Figure S2). We computed axes using whole genome shotgun sequencing data from present-day Dai (southern China), Nasioi (northern Solomon Islands), and New Guineans (from the Eastern Highland and Middle Sepik areas of Papua New Guinea) and projected other individuals. The first principal component (PC) correlates with the proportion of East Asian-associated ancestry (PC1; lower on left, higher on right), while the second PC differentiates Papuan ancestry from the Solomon Islands and New Guinea (PC2; up to down). A tight cluster of Palauans, including the ancient individuals from Koror Quarry, Ucheliungs, Omedokl, and Ngkeklau, as well as present-day individuals, is located along the genetic cline linking groups from New Guinea and other Micronesians (Figure S2).

**Figure 2.**
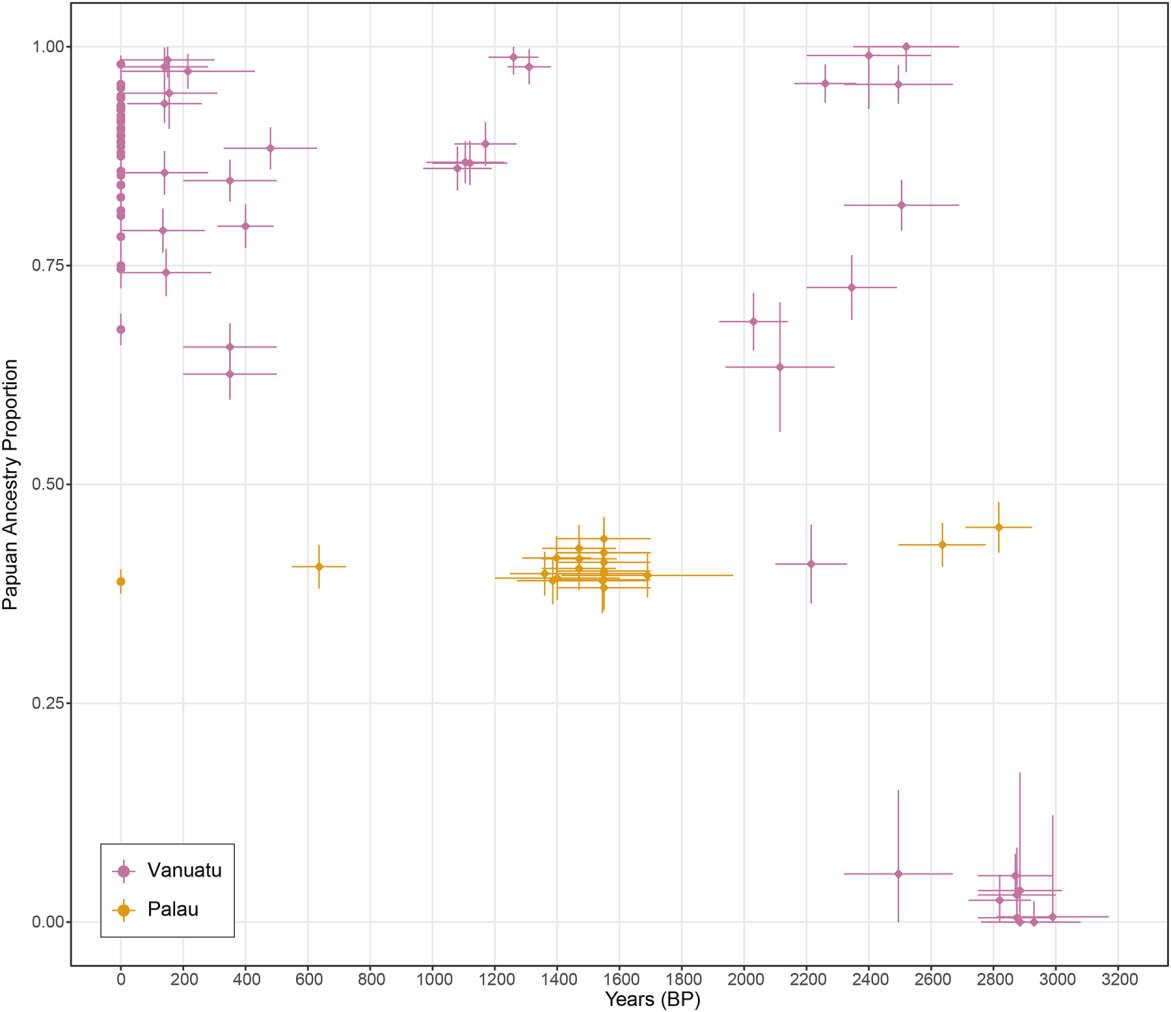
Papuan ancestry proportion in Vanuatu and Palau over time. Estimates for newly reported Palauans are from two-component *qpAdm* models; for Vanuatu individuals from the literature^2–5^. Vertical bars show 95% CI of Papuan ancestry proportions for each individual (ancient) and population (present-day), truncated at 0 and 1. For ancient individuals, we also use horizontal bars to show the uncertainties (95% CI) of the dates.

We computed the symmetry statistic *f*_4_(X, Kankanaey; New Guinea Highlanders, Dai) and confirmed that all the ancient individuals from Palau had significant Papuan admixture (using Kankanaey as a baseline with no evidence of Papuan ancestry) (Table S4.1). Using an *f*_4_-statistic-based regression approach that estimates affinity to different Papuan sources after correcting for biases due to East Asian admixture (Table S4.2 and Figure 1B), we find that the Papuan admixture is more closely related to people of the New Guinea Highlands than to people from the Solomon Islands or the Bismarck Archipelago (Figure 1B), corresponding to approximately 40% genetic ancestry with very small standard errors (Table S3.4). This estimate is very similar to the level of Papuan ancestry in present-day Palauans inferred by the same methodology^6^. The patterns in Palau differ qualitatively from those in the Southwest Pacific: Palau shows a relatively stable level of Papuan ancestry over the past 2800 years, whereas Vanuatu exhibits a sharp increase following the end of the Lapita period around 2500 BP (Figure 2).

Using *qpAdm* we estimate that Papuan ancestry in individuals from Ucheliungs (43.2% ± 0.9%) was slightly higher than in Koror Quarry (40.7% ± 0.7%), Omedokl (40.0% ± 0.9%), and Ngkeklau (39.6% ± 0.8%) (Table S3.4). We computed the statistic *f*_4_(X, Ucheliungs; New Guinea Highlanders, Dai), and confirmed a small but statistically significant excess of Papuan ancestry in Ucheliungs compared to Koror (|Z| = 2.849 standard errors above zero), Omedokl (|Z| = 3.524), and Ngkeklau (|Z| = 3.708) (Table S4.1). We also grouped these individuals based on the ranges of their radiocarbon dates (Table 1), corresponding to 2900–2500 BP, 1700–1200 BP, and 700–500 BP, respectively. The proportions of Papuan ancestry in Palau decreased up to 10% over time using individuals from Ucheliungs (2900–2500 BP) as a baseline (Table S3.5), and we confirmed with *f*_4_-statistics that this decrease was statistically significant (|Z| = 2.8 to 5.5) (Table S4.1). For all sites as in present-day people^6^, the Papuan ancestry in Palau is inferred to be primarily derived from male ancestors, with significantly more Papuan ancestry on the autosomes than on the X chromosome (|Z| = 1.8 to 4.1) (Table S3.6).

Late Unai and Latte individuals from Guam, and Lapita people from Vanuatu and Tonga, consistently match the East-Asian ancestry in prehistoric Palau, but one was not a significantly better proxy than the other (Table S3.4). To test formally for a distinctive FRO_Palau_ lineage in prehistoric Palauans, we refitted the admixture graph as shown in Figure S3, now using ancient DNA data to represent Palauans. The best-fit model inferred prehistoric Palauans to have approximately 60% FRO_Palau_ and 40% Papuan ancestry similar to present-day Palauans, providing evidence of substantial genetic continuity for at least 2800 years in Palau.

### Papuan and East Asian ancestry in Palauans admixed before the initial peopling of Palau

**Figure 3.**
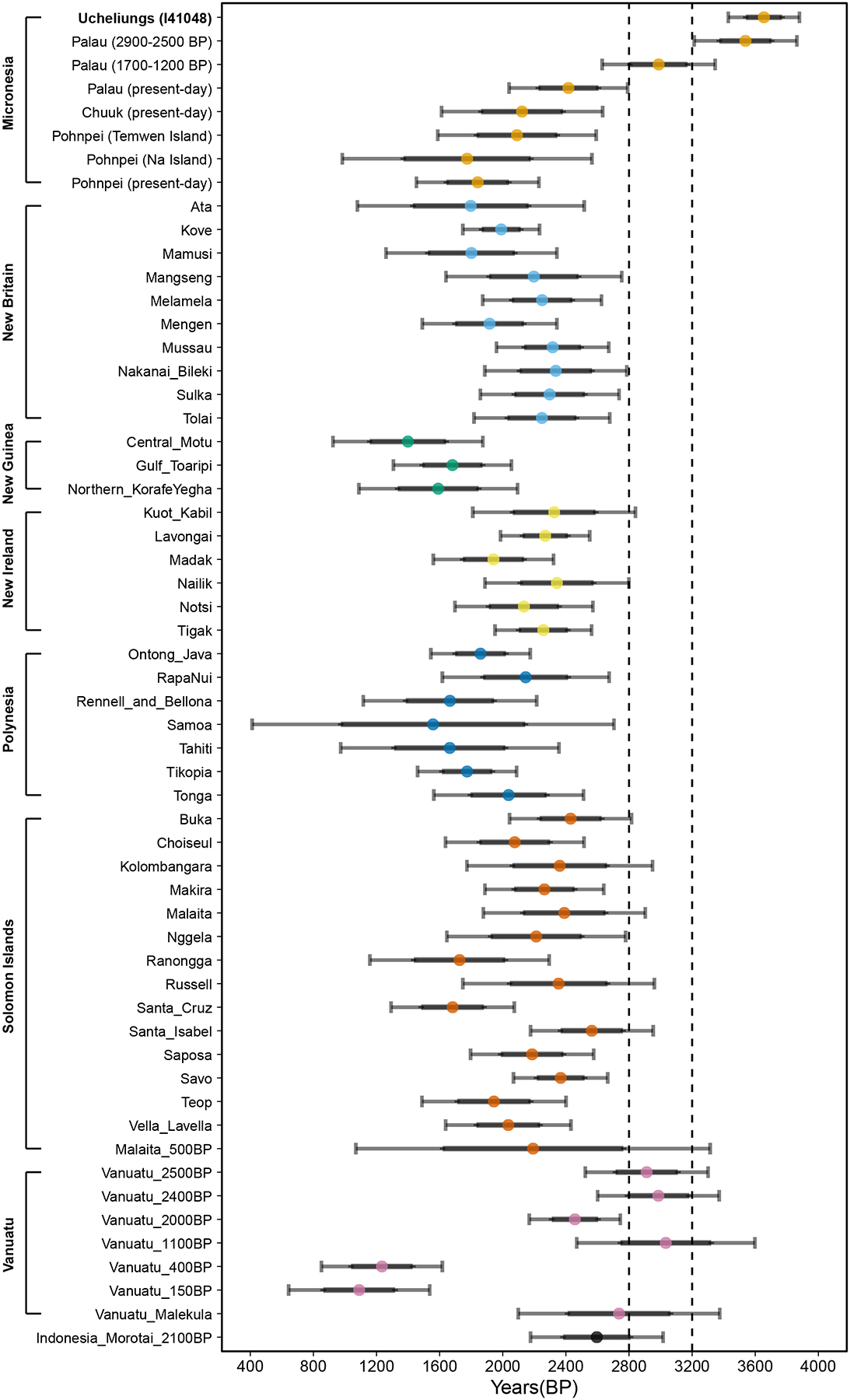
Admixture date estimation for diverse populations from the Pacific. Thick and thin error bars correspond to one standard error of the estimate and the 95% confidence interval (CI), respectively. Two dashed lines represent the early settlement period of Palau (3200–2800BP).

Using DATES, we estimate that the average time of admixture between the lineage of FRO_Palau_ and Papuan_NewGuinea_ is 30.1±3.5 generations (95% CI of 37–23 generations) before the time when the most ancient Palauan I41048 lived (Table S3.7). Based on anthropological and genetic research^19^, we assume an average of 28.4±0.7 years per generation (95% CI of 27–30 years). We further incorporate the direct date range from the oldest Ucheliungs individual (I41048) after calibrating: 2799±53 calBP (95% CI of 2924–2710 calBP) (Table S3.1). Propagating all three standard errors (Table S4.3), we find that the 95% CI of the admixture time is 3880–3429 BP (Table S3.7). Palau is generally viewed as being occupied 3200–3000 BP based on radiocarbon dates from Ulong Island^20,21^. Combining this archaeological inference with our genetic data, we conclude that the ancestors of the first people of Palau were already mixing with people of Papuan ancestry close to a millennium before the ~2500 BP large-scale movement of Papuan ancestry into the Southwest Pacific (Figure 3). The second-oldest Ucheliungs individual (I41049) has a radiocarbon date uncertainty range overlapping the oldest individual. When the analyses for these two individuals are combined, we still obtain a date of admixture of 3864–3213 BP, older than the archaeologically inferred peopling of Palau (Table S3.7). Our estimates of admixture time for the more recent Palauan individuals have 95% confidence intervals that in a number of cases also definitively imply an admixture date >3200 BP, that is, pre-colonization (Table S3.7), although some confidence intervals also include dates after 3200 BP. For present Palauans the admixture date estimate is significantly more recent at 2791–2040 BP (Table S3.7), which could be explained by continuing migration into Palau after 3200 BP and indeed would be consistent with the higher proportions of East Asian ancestry in later individuals (Table S3.5).

Our key finding of major Papuan-East Asian admixture prior to the initial settlement of Palau is dependent on the date of the oldest Ucheliungs individuals, and we therefore explored the effect of alternative assumptions for calibrating the radiocarbon date of the oldest Ucheliungs individual (I41048). Specifically, we explored marine reservoir corrections assumptions (ΔR) of −250±50 years to +168±43 years used in the literature, as an alternative to the −140±35 year estimate^21^ available for Palau based on the Marine20 database, which we use as the basis for the calibrations quoted in the main text. More positive ΔR values yield more recent calibrated dates, whereas more negative ΔR values result in older dates; ΔR estimates that are specific for Palau’s southwest lagoon where Ucheliungs is found are all in the negative part of this range: −250±50 years^22^, −140±35 years^21^, and −52±22 years^23^, which predict older dates. If we assume a marine proportion in ancient Palauan dietary protein of 50%, which makes our date estimates conservatively more recent than they would be if we used the estimate of 25% marine dietary protein from the ~3100 year old burials at Chelechol ra Orrak limestone rock shelter (Site IR-1:17)^24^, the 95% confidence intervals for the Papuan-East Asian admixture date are always older than around 3200 BP regardless of the assumed ΔR (Tables S3.3 and S3.7). The confidence intervals for admixture are also always older than around 3200 BP using the converse approach of setting ΔR of −140±35 years^21^ and varying the assumed proportion of marine protein in the diet from 0% to 100% (Tables S3.3 and S3.7).

Data from Chelechol ra Orrak are consistent with our results from a genetic perspective too. Although no genome-wide data are available from this site and we were not able to analyze samples from it, four mitochondrial genome sequences were recovered from seven specimens (2717–1566 calBP) and belonged to the B4, D4, E1, and M7 haplogroups, all common in East Asia^18^. The mitochondrial haplogroups that we newly report include E1/E2, B4/B5, and P1 (Table 1), of which E1/E2 and B4/B5 are common in East Asian populations (like the pattern in Orrak). The P1 haplogroup is mainly found in Papuans, Melanesians, and Indigenous Australians, showing that the Papuan ancestry in Palauans arrived on both the maternal and paternal lineages. All these haplogroups are also seen in present-day Palau^6^.

### A plausible Wallacea source for the ancestors of Palauans

To obtain insight into the origins of the earliest inhabitants of Palau who most likely had both East Asian and Papuan ancestry, we co-analyzed our data with previously reported data from neighboring regions. We find that the East Asian ancestry in Palauans (FRO_Palau_) is more closely related to the East Asian ancestry in 2100-year-old Eastern Indonesians from Aru Manara and Tanjung Pinang on Morotai Island, Northern Maluku^25^, than to the early FRO lineages from the southwest Pacific and the Marianas (Table S3.8). Thus, the distinctive combination of FRO_Palau_ and Papuan_NewGuinea_ is uniquely shared between Palau, and these Morotai Island individuals. When we use ancient Morotai as a source in *qpAdm* to model the ancestry of Palau, models succeed even with early individuals from Vanuatu and Tonga (FRO_SouthwestPacific_) or early individuals from the Mariana Archipelago (FRO_Marianas_) in the outgroups. This further supports the conclusion that the particular admixture of East Asian and Papuan ancestries in the early ancestors of the Palauans also occurred in the ancestry of people in eastern Indonesia (Figure S3). Due to limited sampling, we cannot rule out that other eastern Indonesian regions had similar ancestry and could have been sources.

The 2100-year-old Morotai samples harbored about 30% Papuan ancestry from New Guinea of the same type as in Palau, and 70% East Asian ancestry^25^ (10% more East Asian than Palauans). We estimate that the admixture of the two ancestries in Morotai individuals occurred approximately 23±3 generations before the time these individuals lived (Figure S4). By incorporating the calibrated direct radiocarbon dates, we calculated the 95% CI of the admixture time in Morotai individuals to be 3016–2176 BP (similar to an earlier estimate^25^), and later than that in the earliest Palauans, potentially because the ancient Morotai individuals continued to receive East Asian ancestry over time, as did later Palauans.

## DISCUSSION

The settlement of Palau was one of the earliest events in the dispersal of humans to Remote Oceania. Previous reconstructions of ancient and modern DNA revealed male-biased mixing of First Remote Oceanian and Papuan lineages between 2500 and 2000 BP in two regions of Remote Oceania (the southwest Pacific and central Micronesia), as well as in the genetic ancestry of present-day Palauans^2,5,6^. A natural expectation based on these patterns was that the admixture of Papuan ancestry into Palauan ancestors occurred around the same time as in other Remote Oceanians, so our new results of earlier Papuan mixture were surprising^2–6,8^.

What was the geographic source of the FRO_Palau_ ancestry, which our analysis suggests was the first splitting of First Remote Oceanian lineages? An earlier study estimated that the FRO_Palau_ lineage admixed in the Marianas between 2400 and 1700 BP, giving rise to the ancestors of Latte Period population but without the Papuan ancestry present in Palau^6^; thus, it is unlikely that this ancestry originated directly from Palau. Given the similarity between the Papuan-FRO ancestry admixture observed in Palau and that found in 2100-year-old individuals from Morotai Island in the Northern Maluku, it is plausible that the source instead lay somewhere in the Maluku region of northern Wallacea. However, Morotai people speak a Papuan language not an Austronesian language as do Palauans^26^; and archaeological studies indicate no pottery-using population present in Morotai until 2300–2000 BP, when a strong Metal Age influence emerged at the same time as the Papuan_NewGuinea_-FRO_Palau_ mixture occurred. The genetic date of admixture in Morotai postdates that in Palau. Therefore, the Morotai population today cannot be a direct descendant without admixture of the first peoples of Palau.

The broader region of eastern Indonesia including Maluku is a prime candidate for the source of both FRO_Palau_ ancestry and the mixed Papuan-East Asian population that spread into Palau after 3200 BP. Pottery-using populations existed as early as 3500 BP in the north arm of Sulawesi and Halmahera^27^. Around this time, sea level was declining from its mid-Holocene highstand, a long-term process that changed marine habitats and widened shorelines where arable lowland soils began to accumulate. At the same time, increasing variability in the El Nino-Southern Oscillation weather system brought stronger monsoons and more frequent droughts^28^. These environmental changes may have prompted mobile marine foragers from northern Wallacea to explore the uninhabited small island groups of Western Micronesia such as Palau, where highly productive shallow marine habitats were emerging^29^. Ancient voyaging models incorporating seafaring simulation techniques with analyses of climatic and oceanographic data and island distributions make it plausible that people departing from the arc from Mindanao to Halmahera, including northern Sulawesi, could have made successful landfalls in Palau^30,31^. Scenarios of Palau’s settlement from northeastern Indonesia would also be consistent with similarities found between the rock paintings of Palau and the islands of Maluku^32^, which both rely on red pigment and are placed on highly visible coastal cliffs. These scenarios could be tested with ancient DNA data from the relevant times and places.

It is tempting to hypothesize that the initial settlers of Palau came from the Admiralty Islands like Manus, which have been identified as a potential source region for the Papuan ancestry in Central Micronesia, and which have a similar type of Papuan ancestry as the people of Palau^6^. However, such a scenario cannot explain our data, as the East Asian ancestry in Palau and the Admiralties are demonstrably different. While the East Asian ancestry found in the Southwest Pacific and Central Micronesia is generally “Lapita-like” (FRO_SouthwestPacific_)^6^, the East Asian ancestry in Palau is distinctive (FRO_Palau_), and so the East Asian–Papuan mixture histories in the two regions are unrelated.

These findings qualitatively change our understanding of the first settlement of Remote Oceania. A commonly discussed historical linguistics model proposes a Taiwanese origin for the Austronesian languages and a Pacific-wide dispersal through a long-term demic expansion of Neolithic populations dependent on rice cultivation, from Taiwan to the Philippines, and onward into western Micronesia, including the Marianas and Palau. However, our findings contradict this model in Palau, showing that the initial settlement of Remote Oceanic islands was not simply the result of a direct migration from Taiwan through the Philippines with minimal interaction or admixture with local populations. In particular, we show that the path to Palau corresponding to the first branching FRO lineage followed a more complex pattern, incorporating alternative routes through Eastern Indonesia and long-term interactions between ancient Papuan and East Asian populations.

### Limitations of the study

An open question is the geographic and temporal origin of the three distinct First Remote Oceanian lineages, especially FRO_Palau_. Ancient DNA research focusing on individuals from earlier periods (pre-2800 BP) from Palau, the Philippines, Sulawesi, and Halmahera, would provide critical insight into demographic shifts before and after the Austronesian dispersal.

## RESOURCE AVAILABILITY

### Lead contact

Further information and requests for resources and reagents should be directed to and will be fulfilled by the lead contact, David Reich (reich@genetics.med.harvard.edu).

### Materials availability

Open science principles require making all data used to support the conclusions of a study maximally available, and we support these principles here by making fully publicly available not only the digital copies of molecules (the uploaded sequences) but also the molecular copies (the ancient DNA libraries themselves, which constitute molecular data storage). Those researchers who wish to carry out deeper sequencing of libraries published in this study should make a request to the corresponding author D.R. We commit to granting reasonable requests as long as the libraries remain preserved in our laboratories, with no requirement that we be included as collaborators or co-authors on any resulting publications.

### Data and code availability

Newly reported ancient DNA data of this study are fully publicly available and have been deposited in the European Nucleotide Archive (ENA project accession number PRJEB96382). This paper does not report original code.

## STAR★METHODS

### EXPERIMENTAL MODEL AND STUDY PARTICIPANT DETAILS

#### Ancient individuals

An extensive description of the archaeological and anthropological context of the ancient individuals analyzed in this study is provided in Supplemental Information.

### METHOD DETAILS

#### Ethical approvals and sampling

In February 2023, co-authors from this study (Joanne Eakin and Jolie Liston) met with representatives of the Palau Bureau of Cultural and Historic Preservation (BCHP) in Ngerulmud, Babeldaob, Palau, to finalize a memorandum of agreement (MOA) for ancient DNA analysis of skeletal remains housed at the BCHP storage facility and the Belau National Museum in Koror, Palau, which was approved by Kiblas Soaladaob, Director of the BCHP and co-author, on February 7, 2023. With the assistance of BCHP personnel, we inspected skeletal remains and evaluated elements for sample selection. To maximize DNA retrieval success rates and minimize damage to skeletal remains, we collected petrous bones whenever possible (otherwise molar teeth). We collected 32 samples from three archaeological sites: 22 from the Ngermereues Ridge burial cave (Site OR-3:30) at Ngesaol, Koror (Koror Quarry), six from the Ucheliungs Rock Island burial cave (Site OR-14:8) (Ucheliungs), and four from the Omedokl Rock Island burial cave (Site OR-15:8) (Omedokl). With the assistance of Belau National Museum personnel, we also collected samples from the skeletal remains of five individuals at the Museum’s storage facility in Koror. These specimens lacked archaeological documentation but were believed by museum staff to be from the Ngkeklau area of northeastern Babeldaob (Ngkeklau). Despite their imprecise provenance, we decided to include them in our study, and we obtained genome-wide data and direct radiocarbon dates from these individuals, which provide useful datapoints in our analysis.

On February 13, 2025, the results of this study were presented to Palau High School students and Palau community representatives by a team of five authors, two of whom participated in person and three by video link. Audience feedback from this presentation was incorporated before submission. While it is not always possible to gather input from everyone with a connection to an ancient culture, consultation informs the narrative, and promotes sensitivity to stakeholder perspectives.

#### Archaeological context of the samples analyzed in this study

Palau’s occupation sequence may be divided into four loosely defined phases or periods (Table S1 and Supplemental Information): Colonization Period 3200–2800 BP, Expansion Period c. 2800–2400 BP, Earthwork Period c. 2400–1100 BP, and Stonework Period c. 1100 BP to Western contact in 1783 (after Liston 2009)^33^. Of the eleven calibrated radiocarbon dates, one corresponds to the end of the Colonization Period and beginning of the Expansion Period, one to the Expansion Period, eight to the Earthwork Period, and one to the Stonework Period.

#### Wet laboratory work

The skeletal samples were processed into powder at the University of Vienna, Austria. Each sample was processed in a clean room suite, where several measures were used to reduce the presence of contaminating modern DNA. The room is under positive air pressure to minimize the introduction of external contaminants and is sterilized with UVC light to destroy modern DNA. While in the clean room, trained ancient DNA technicians wear a coverall, two layers of gloves, and a facemask with a visor to ensure the samples are exposed to minimal amounts of their own DNA.

We extracted powder from petrous bones using a fine sandblaster to clean the superficial surface exposed to human contact and the depositional environment and remove the spongy bone surrounding the cochlea^34,35^. After exposing and cleaning the cochlea, we milled half or all of it into a fine powder in a mixer mill. For tooth samples, we first cleaned the surface of the roots with a sanding disk to remove contaminants present on the outer layer. We then drilled into the base of the roots with a dentistry drill equipped with a previously unused standard drill bit. We did not drill into the crowns. We aimed to include 37mg per ancient DNA extraction.

Subsequent wet laboratory steps were all carried out at Harvard Medical School, USA (Tables S2.1 and S2.2). To extract DNA, we dissolved bone powder in an extraction solution, and then used published protocols to isolate and clean up the extracts, and to convert them into individually indexed libraries using protocols optimized for ancient DNA molecules which are typically damaged, short, and present in low concentrations^36,37^.

Because ancient samples typically have very low percentages of human DNA (usually less than 10%), direct sequencing can be prohibitively expensive. Even for samples that have relatively high proportions of human DNA, as is often the case with petrous bone samples, the great majority of sequences do not overlap the positions in the genome that are most informative and for which data on other individuals are available, which also increases sequencing costs. To address this challenge, we generated data using a strategy that enriches ancient DNA libraries for specific regions of interest in the genome^38^; in this case around 1.35 million SNPs directly targeted by the “Twist Ancient DNA” in-solution enrichment reagent. Including SNPs near the targeted ones for all the newly reported individuals, we analyzed data from up to about 2 million polymorphic SNPs. We sequenced the enriched products with Illumina instruments.

#### Bioinformatic processing of raw data

Once genetic data were generated, we assigned reads to libraries according to their barcodes and indices, allowing at most one base pair (bp) mismatch of the barcodes for each pair of reads. After trimming barcodes and adapters, we merged forward and reverse reads of each pair, requiring a minimum of 15-bp overlapping sequence and a maximum 1-bp mismatch. We then mapped all sequences to the mitochondrial reference genome RSRS and the nuclear reference genome (GRCh37 / hg19). Before variant calling, we kept one representative each for sequences aligned to identical outer coordinates, which are likely to be PCR duplicate copies of the same original ancient molecule. We also only analyzed sequences that mapped to the reference genome uniquely. We applied filtering with a mapping quality threshold of no less than 10. We generated pseudo-haploid genotypes for genomic analyses by assigning the observed base to one randomly chosen sequence covering each targeted SNP position. The computational pipelines with specific parameters used in the bioinformatic processing are publicly available on the GitHub platform at https://github.com/dReichLab/ADNA-Tools and https://github.com/dReichLab/adna-workflow.

After bioinformatic processing, we assessed the authenticity of ancient DNA data through several criteria (Tables S2.1 and S2.2). We measured cytosine-to-thymine substitution rates at the terminal positions of the sequences, since characteristic ancient DNA deamination is indicative of the presence of authentic ancient DNA. We only used libraries for which the number of human sequences was at least an order of magnitude above the background level in extraction and library controls. For all ancient DNA data, we inferred genetic sex by testing whether the ratio of reads mapping to X and Y chromosomes was consistent with the sequences deriving entirely from a male or a female. We determined potential contamination from external DNA by (a) computing the percentage of mitochondrial fragments matching to the reference sequence of the consensus mitochondrial haplogroup for each individual^39,40^, and (b) measuring the rate of heterozygous sites on the X chromosome for males (assumed to have only one X chromosome) compared to the expectation for a female^41^. We determined mitochondrial haplogroups using HaploGrep2^42^, and Y-chromosomal haplogroups by comparing SNPs (using all reads) to the International Society of Genetic Genealogy Y-tree (http://www.isogg.org). We searched for newly reported samples that were genetically identical and closer (up to third-degree relatives) using READv2^43^. We identified four pairs of genetically identical individuals (detailed below) and no close relatives.

Of 37 skeletal specimens that we collected, 17 teeth and 14 cochlea specimens yielded enough ancient DNA for data quality and authenticity evaluation. Six specimens yielding fewer than 5000 autosomal SNPs were considered to have failed analysis and their data are not reported in this paper. Four specimens (I41044, I41050, I41055, and I43884) were genetically identified as from the same individuals as another four, and we merged the data for each pair of individuals and reported four unique ones (I41043, I41049, I41054, and I43894). One specimen (I43046) yielded good-quality data but had indications of marginal levels of contamination, and we therefore restricted the analyses to sequences with evidence of characteristic ancient DNA damage. We fully reported and analyzed the genome-wide ancient DNA data of this sample with the other 20 samples passing our authenticity evaluation and that yielded data from between 10,924 and 1,826,120 autosomal SNPs from the set of ~2 million in our main analyses. Among these 21 acceptable samples, nine were from teeth, consistent with previous findings that ancient DNA analyses, especially in warm climates, are more successful for petrous bones than teeth.

#### Direct radiocarbon dating and calibration

The radiocarbon age of the global ocean (R) is older than the atmosphere by hundreds of ^14^C years. A recent revision of the marine mixing model and calibration curve^44^, Marine20, estimated a value of R to be ~550 ^14^C years older than previous estimates^45–47^. There are also secular differences in the apparent ^14^C age of marine organisms due to local offsets (ΔR)^48^. Away from the continental margins that are subject to upwelling and other circulation processes, Pacific Basin surface waters are generally better mixed with the atmosphere than the average global ocean, leading to negative values for ΔR. Calibrations for each burial date were performed in OxCal v.4.4^49^ as mixtures of terrestrial (IntCal20) and marine (Marine20) assuming around 50% of marine carbon contributing to the dietary protein of prehistoric Palauans based on a previous estimation from Stone et al. (2020)^18^, with an uncertainty of ±10%, and using the recalculated ΔR = −140 ± 35 ^14^C years^21^ for Palau from the Marine20 database (http://calib.org/marine/). We use the standard definition of 1950 Common Era (CE) as 0 calBP. For comparison, we repeated calibrations assuming 0%, 20%, 50%, and 100% marine dietary protein (Tables S3.1 and S3.3).

We submitted 12 samples to the Center for Applied Isotope Studies, University of Georgia (UGA), to obtain direct radiocarbon dates for our newly reported samples. Because most samples are subject to some degree of alteration or contamination, effective pretreatment is essential for accurate radiocarbon dating^50^. Contaminants are defined as exogenous organic carbon-containing compounds. Pretreatment aims to isolate and remove these prior to measurement. All the samples underwent an initial physical examination to assess composition and preservation and then physical and chemical pretreatments. For ancient bones/teeth, this includes demineralization and gelatinization, followed by filtration through a particle filter to remove detritus, with the freeze-dried collagen then subjected to radiocarbon dating. However, this method may yield unreliable dates. Over time, ancient bone can become contaminated with exogenous organic carbon molecules of varying ages that bind to the collagen within the bone’s hydroxyapatite. The resulting radiocarbon dates may be significantly skewed because particle filters cannot remove these chemically bound contaminants. In some environments, the contamination may even be older or roughly the same age as the sample, but there is no definitive way to determine this without directly dating the contaminating humic acids.

We processed five additional samples through the Center for Stable Isotopes (CSI) at the University of New Mexico which we then measured with AMS at Penn State University’s Radiocarbon Laboratory (PSUAMS). At CSI, we used one of two sample preparation methods depending on the characteristics of the samples. Ultrafiltration, as applied to four samples in this study, is based on the assumption that, at low temperatures (~70 °C), solubilized collagen (hydrolysate) molecules larger than 30,000 Daltons (30 kDa)—approximately one-third the mass of the non-cross-linked chains of the collagen type I heterotrimer (comprising two α1 chains and one α2 chain)—originate from bone collagen, whereas smaller molecules (<30 kDa) are presumed to consist of non-collagenous organic contaminants unsuitable for dating. However, in challenging environments such as the tropics, where collagen is often already severely degraded, both damaged collagen fragments and contaminants can pass through the 30 kDa membrane, undermining the effectiveness of this method. In such cases, collagen purification can be achieved using XAD-2 resin purification, as applied to one sample in this study. This method involves hydroxylating the collagen at 110 °C to ensure completely cleavage of peptide bonds, then passing the hydrolysate through a non-polar hydrophobic XAD-2 resin in a solid-phase extraction column. The resulting eluate, primarily composed of amino acids, is freeze dried and prepared for combustion, graphitization, and radiocarbon dating following standard protocols. The resulting 17 dates were assessed for their chronological consistency (Table S3.1), and cross-validated them with previously reported dates (Figure S1 and Table S3.2), resulting in 11 reliable new dates.

### QUANTIFICATION AND STATISTICAL ANALYSIS

#### Population structure overview

To visualize population structure, we carried out Principal Component Analysis (PCA) using *smartpca* in EIGENSOFT^51^ v.7.2.1 using default parameters with three options: “lsqproject: YES”, “autoshrink: YES”, and “inbreed: YES”. We combined genotype data from 1,247 previously published ancient and present-day individuals from Oceania and Southeast Asia (for a complete list of reference populations, see Liu et al. 2022)^6^, along with 21 newly reported ancient individuals from Palau. These individuals were genotyped using different strategies, including the Affymetrix Human Origins SNP array, the GenoChip 2.0 microarray, the Multi-Ethnic Global Array, the 1240K Agilent in-solution enrichment reagent, and Twist ancient DNA. To maximize the number of SNPs included in the principal component analysis (PCA) and to improve resolution of genetic relationships among groups^6^, we performed PCA on the union of SNPs across these five platforms, totaling approximately three million autosomal SNPs. PCs were defined using whole-genome shotgun data from four present-day populations—Papuans from the Eastern Highlands and the middle Sepik region of New Guinea, Nasioi speakers from Bougainville Island in the Solomon Islands, and Dai individuals from southern China—to minimize the influence of genetic drift across Oceanian and East Asian populations. Other prehistoric and present-day individuals were then projected onto the first two principal components, generating a two-dimensional plot (Figure S2). One individual (I43046) appeared to be separated from others from the same site (not shown in Figure S2), likely due to a low number of analyzed SNPs. This outlier was excluded from subsequent analyses.

#### Analyses of genetic ancestry

To detect Papuan admixture in Palau, we computed *f*_4_-statistics using *qpDstats* in ADMIXTOOLS^52^ v.7.0 with the parameters “f4mode: YES” and “printsd: YES”. We computed standard errors using a block jackknife. We computed *f*_4_(X, Kankanaey; New Guinea Highlander, Dai), using New Guinea Highlanders as a representative population with unmixed Papuan ancestry and Kankanaey as a representative population related to the ancestors of East Asians without Papuan admixture. Results are summarized in Table S4.1.

To trace the deeper origin of Papuan ancestry in ancient Palau, we used an *f*_4_-statistic-based regression approach^9,10^ to distinguish differential sources that we hypothesized might have contributed to ancient Palauans (Figure 1B). We computed all *f*_4_-statistics using *qpDstats* in ADMIXTOOLS with the parameters “f4mode: YES” and “printsd: YES”. We carried out regression using the inverse-variance-weighted least-squares method as implemented in R v.3.6.2. We computed residuals for each data point and their standard errors through block jackknife resampling using *qp4diff* in ADMIXTOOLS. We computed residual Z scores as the residuals divided by their standard errors. We restrict analyses to ~169,000 autosomal SNPs that form the intersection between the core set of 1.15 million autosomal SNPs targeted by the 1240k in-solution enrichment reagent, and the MEGA array. All statistics are in Table S4.2.

#### Reconstruction of genetic models

To infer admixture proportions, we used the *qpfstats* and *qpAdm* methods^53^ in ADMIXTOOLS, determining proportions of Papuan ancestry fitting two-way mixture models for Palauans. When running analyses with *qpAdm*, we first ran *qpfstats* using “allsnps: YES” and “inbreed: YES”. We used the *qpfstats* output as the *qpAdm* input to perform the modeling. For all models, we interpreted *P*-values higher than 0.05 as statistically acceptable fits. We also used *qpAdm* and *f*_4_-statistics to compare if the individuals from Ucheliungs had more Papuan ancestry than other prehistoric and present-day Palauans, and to test and verify the genetic link between ancient people from Palau and Morotai. We restrict our analyses to 1240K SNPs, and results are summarized in Figure 2 and Tables S3.4, S3.5, S3.6, and S3.8.

We used *qpfstats* and *qpGraph* in ADMIXTOOLS to fit admixture graphs. We first ran *qpfstats* using “allsnps: YES” and “inbreed: YES”, and then used the output of *qpfstats* as the input to *qpGraph*. We restrict analyses to 1240K SNPs and show results in Figure S3.

#### Estimation of admixture dates

To estimate the dates of admixture events, we used the Distribution of Ancestry Tracts of Evolutionary Signals software (DATES v.3530)^54,55^. We applied the analyses to the ~2 million SNPs set to maximize statistical power. As a proxy for the East Asian source, we merged five Ami/Atayal/Kankanaey individuals from Taiwan and the Philippines, nine Dai individuals from South China, and 42 Han individuals from mainland China. As a proxy for the Papuan source, we merged eight New Guinea Highlanders, six Papuans from the Middle Sepik region of the island of New Guinea, and 11 Nasioi individuals from the Solomon Islands. We also merged individuals from Palau of similar dates, especially the large number of samples dating to around 1700–1300 BP from Koror, Omedokl, and Ngkeklau. This strategy allows us to use the maximum number of SNPs for each analysis and increase the sample size of both the reference and target populations, increasing statistical precision. We ran DATES with ‘maxdis: 1.0’ to ensure we can detect even relatively long blocks of linkage disequilibrium (LD) of the type that would be expected to be generated by relatively recent admixture events. For the ranges of admixture dates (95% CI) reported in Figure 3, Figure S4, and Table S3.7, we propagated three sources of uncertainty for our estimation: the genetic uncertainty inferred by DATES, the radiocarbon date uncertainty, and the generation interval uncertainty, as detailed in Table S4.3.

Given the wide range of marine reservoir corrections (ΔR) reported for Palau, we calibrated the radiocarbon date for the oldest Ucheliungs individual (I41048) using a range of ΔR values averaged across the archipelago and incorporated varying proportions of marine dietary protein contributions in the calibration process (Table S3.3). In general, more positive ΔR values yield more recent calibrated dates, whereas more negative ΔR values result in older dates. Similarly, lower estimates of marine dietary protein contribution tend to produce older corrected dates, while higher marine input leads to younger corrected dates. We show that the estimation of admixture timing for I41048 under alternative radiocarbon calibrations are consistently earlier than 3200 BP, when the marine dietary protein contribution to the diet of ancient Palauans is less than or equal to 50%. We used a mixed calibration curve combining 50% IntCal20 and 50% Marine20 with a 10% variance as suggested in Stone et al. (2020)^18^ and applied a ΔR value of −140 ± 35 ^14^C years.^21^.

#### ROH and IBD calling

We identified Runs Of Homozygosity (ROH) within our ancient DNA dataset using the Python package hapROH v.0.3a4 (https://pypi.org/project/hapROH/). As recommended by this method for the 1240K SNP set, we only included ancient individuals with at least 400,000 covered SNPs. We ran the method to identify ROH >4 centimorgans (cM) using the default parameters, which were optimized for the ancient DNA 1240K data. We separated the inferred ROH into four categories in length: >4 cM, >8 cM, >12cM, and >20 cM. We report the total sum of ROH for each category in Table S2.3. The samples were imputed using the software GLIMPSE according to the standard processing steps. Each sample was imputed separately to avoid batch effects. We used the phased haplotypes from the 1000 Genomes dataset as a reference panel for imputation. The details of the imputation steps are described elsewhere^56^. We then used the imputed and phased data in the software ancIBD (https://github.com/hringbauer/ancIBD). As recommended in ancIBD manual, we restricted our analyses of IBD calling to the 1240K SNP set, for which ancIBD is optimized. We only included ancient individuals with at least 400,000 covered SNPs for reliable IBD calling. We then followed the instructions of ancIBD to detect IBD segments of length ≥8cM. The summary statistics for each pair of individuals with IBD detected are shown in Table S2.4.

## Supplementary Material

DocumentS1_Supplemental_Information **Document S1**. Archaeological context, Tables S1 and S3, and supplemental references

FigureS1_Chronology **Figure S1. Chronology for the sites reported in this study, related to** Table 1. Eleven newly reported radiocarbon dates in this study (dark grey) are plotted with 18 previously published dates (14 of human bones and four of marine shells) from four sites. The sources of published data are summarized in Table S3.2 and Supplemental Information. The date ranges shown in this figure represent the 95.4% confidence intervals of the calibrated dates. We recalibrated the published dates. For human bones, we calibrate these dates based on a mixed calibration curve combining 50% IntCal20 and 50% Marine20 (ΔR=−140±35 ^14^C years) with 10% variance. For marine shells, we calibrate the dates based on Marine20 with a ΔR=−140±35 ^14^C years. Each date is labeled using the AMS lab code as the primary identifier, followed by the specimen code in brackets. If either piece of information is unavailable for a given published date, the available identifier is used, with specimen codes consistently enclosed in brackets.

FigureS2_PCA **Figure S2**. **Principal components analysis (PCA), related to** Figure 1. We performed PCA on a unified set of approximately three million SNPs by integrating diverse Pacific populations genotyped on different SNP arrays (Method Details). We computed axes based on whole-genome sequencing data from three present-day populations: Papuans from the Eastern Highlands and the middle Sepik region of Papua New Guinea, Nasioi speakers from Bougainville Island in the Solomons archipelago, and Dai people from Southern China. We projected all the prehistoric and present-day individuals. For the full population list, see^6^.

FigureS3_Admixture_Graph **Figure S3. Fitting ancient Palau using *qpGraph*, related to** Figures 1 and 2. A) Consistent with the previous model fitted to present-day Palauans, ancient Palauans can be fit as a mixture of ancestry related to New Guinea Highlanders and the East Asian lineage (FRO_Palau_) that contributed to the Latte individuals in the Marianas. The maximum residual is |Z| = 2.953, which is consistent with being a good fit after correcting for the number of hypotheses tested. B) We tried modeling ancient Palauans as a mixture of ancestry related to New Guinea Highlanders and the FRO_SouthwestPacific_ lineage. The maximum residual |Z| = 11.284 for this graph, demonstrating poor fit. C) We tried modeling ancient Palauans as a mixture of ancestry related to New Guinea Highlanders and the FRO_Marianas_ lineage. The maximum residual |Z| = 22.221 for this graph, demonstrating poor fit. D) to F) Different admixture graph fits show a genetic link between ancient Palau and Northeastern Indonesia, with the maximum residual |Z| = 1.202, 1.202, and 1.205, respectively. All the models are constructed based on the autosomal intersection between the 1240K and the Twist reagents after removing sites in CpG dinucleotides (i.e. ~826,000 SNPs). Branch lengths are shown in units of average squared allele frequency divergence (multiplied by 1000, rounded to the nearest integer).

TableS2_Samples_and_Libaries **Table S2**. Information for sample and sequencing libraries, related to Table 1

TableS4_F4stats_and_AdmixtureDates **Table S4**. Data for *f*_4_, *f*_4_-regression model, and admixture dates, related to Figures 1 and 3

FigureS4_Admixture_Dates_LD **Figure S4. Linkage disequilibrium (LD) for admixture date estimation, related to** Figure 3. Two-way models for DATES using 56 East Asians and 25 Papuans as proxies to estimate the allele frequencies of the admixing populations. A) LD curve for Ucheliungs individual I41048; B) LD curve for Ucheliungs individual I41049; C) LD curve for the two oldest individuals from Ucheliungs Cave (2900–2500 BP) (N=2); D) LD curve for Omedokl Cave (N=3); E) LD curve for Ngkeklau (N=3); F) LD curve for Koror Quarry (N=11); G) LD curve for 17 individuals who cluster around the same date (1700–1200 BP), including two Omedokl individuals, three Ngkeklau individuals, one Ucheliungs individual, and eleven Koror individuals; H) LD curve for Omedokl individual I41045 (700–500 BP); I) LD curve for ancient Morotai, Indonesia (N=8).

## Figures and Tables

**Table 1.** Newly reported ancient individuals with genome-wide data (N = 21)

Individual ID	Sex	SNPs	Date Range 95.4% CI	Y Haplogroup	MT Haplogroup	East Asian Ancestry	Papuan Ancestry	Locality
I41048	M	1,755,139	2924–2710 calBP	S	P1d1	0.549±0.015	0.451±0.015	Ucheliungs Cave
I41049	M	1,804,743	2776–2495 calBP	O2a2b2a2b	B4b1a2	0.569±0.013	0.431±0.013	
I41056	M	1,306,936	1508–1288 calBP	S	B4a1a1a20	0.584±0.013	0.416±0.013	
I42650	M	74,695	[1700–1400 BP]	BT	B4a1a	0.609±0.018	0.391±0.018	Koror Quarry
I42651	F	62,621	1691–1398 calBP	n/a (female)	n/a (<2x)	0.610±0.019	0.390±0.019	
I42652	M	218,793	[1700–1400 BP]	CF	B4a1a4	0.602±0.015	0.398±0.015	
I42654	F	1,713,739	1695–1414 calBP	n/a (female)	E2	0.604±0.013	0.396±0.013	
I42656	F	1,442,346	1588–1352 calBP	n/a (female)	P1d1	0.573±0.014	0.427±0.014	
I43042	M	456,397	[1700–1400 BP]	S1a1b	B4a1a3a1	0.578±0.014	0.422±0.014	
I43044	F	1,625,251	[1700–1400 BP]	n/a (female)	B4a1a1	0.589±0.012	0.411±0.012	
I43046_d	F	10,924	[1700–1400 BP]	n/a (female)	n/a (<2x)	n/a (too few SNPs)	n/a (too few SNPs)	
I43882	M	1,149,854	[1700–1400 BP]	S1a1	B4a1a4	0.562±0.013	0.438±0.013	
I43883	F	410,077	[1700–1400 BP]	n/a (female)	n/a (<2x)	0.618±0.013	0.382±0.013	
I43894	F	1,749,482	[1700–1400 BP]	n/a (female)	M73a	0.599±0.013	0.401±0.013	
I43895	M	139,589	[1700–1400 BP]	K2	B5b1c	0.578±0.018	0.422±0.018	
I41051	F	1,791,603	1472–1248 calBP	n/a (female)	B4a1a	0.602±0.013	0.398±0.013	Ngkeklau, Ngaraard
I41052	M	1,781,302	1588–1351 calBP	S	B5b1c	0.596±0.013	0.404±0.013	
I41054	M	1,343,729	[1600–1200 BP]	O2a2b2a2b	B5b1c	0.607±0.013	0.393±0.013	
I41042	F	1,489,058	1591–1350 calBP	n/a (female)	E1a1	0.585±0.013	0.415±0.013	Omedokl Cave
I41043	M	1,826,120	1501–1270 calBP	GHIJK	P1d1	0.610±0.014	0.390±0.014	
I41045	F	1,709,935	723–548 calBP	n/a (female)	P1d1	0.594±0.013	0.406±0.013	

**Notes:** Calibrated dates (95.4% CI) are based on a mixed curve combing 50% IntCal20 and 50% Marine20 (ΔR = −140±35 ^14^C years) with 10% variance. Burial context estimates in brackets are derived from direct dates from the same site. MT/Y, mitochondrial DNA/Y haplogroups; SNPs indicate unique autosomal target sites covered at least once and included in the main analyses. Ancestry was estimated by *qpAdm* on autosomal SNPs, using Kankanaey and New Guinea Highlanders as sources. For Individual IDs with “_d”, SNPs were identified exclusively from sequences showing characteristic ancient DNA damage. More information detailed in Tables S2.1 and S2.2.

**Table:** KEY RESOURCES TABLE

REAGENT OR RESOURCE	SOURCE	IDENTIFIER
**Biological samples**		
21 newly reported ancient individuals	This paper	N/A
**Deposited data**		
Sequencing data for 21 newly reported ancient individuals	This paper	ENA accession: PRJEB96382
**Critical commercial assays**		
HiSeq X Ten Reagent Kit v2.5	Illumina	FC-501–2521
NextSeq5 00/550 High Output Kit v2.5	Illumina	FC-404–2002
**Software and algorithms**		
DReichLab/ADNA-Tools	https://github.com/dReichLab/ADNA-Tools	N/A
DReichLab/adna-workflow	https://github.com/dReichLab/adna-workflow	N/A
READ v2	https://github.com/GuntherLab/READv2	N/A
OxCal v4.4	https://c14.arch.ox.ac.uk/oxcal.html	N/A
EIGENSOFT v.7.2.1	https://github.com/DReichLab/EIG	N/A
ADMIXTOOLS v.7.0	https://github.com/DReichLab/AdmixTools	N/A
DATES v.3530	https://github.com/priyamoorjani/DATES	N/A
GLIMPSE2	https://odelaneau.github.io/GLIMPSE/	N/A
hapROH v.0.3a4	https://github.com/hringbauer/hapROH	N/A
ancIBD	https://github.com/hringbauer/ancIBD	N/A
**Chemicals, peptides, and recombinant proteins**		
2X HI-RPM hybridization buffer	Agilent Technologies	5190–0403
Herculase II Fusion DNA Polymerase	Agilent Technologies	600679
Pfu Turbo C Hotstart DNA Polymerase	Agilent Technologies	600412
50% PEG 8000	Anatrace	OPTIMIZE-82100ML
0.5M EDTA (pH8.0)	BioExpress	E177
Sera-Mag^™^ SpeedBead Carboxylate-Modified [E3] Magnetic Particles	Cytiva Life Sciences	65152105050250
silica magnetic beads	G-Biosciences	786–916
10X T4 RNA Ligase Buffer	New England Biolabs	B0216L
Bst DNA Polymerase2.0, large frag.	New England Biolabs	M0537
UGI	New England Biolabs	M0281
USER enzyme	New England Biolabs	M5505
Buffer PB	QIAGEN	19066
Buffer PE concentrate	QIAGEN	19065
1M Tris-HCl (pH8.0)	Sigma Aldrich	AM9856
1M NaOH	Sigma Aldrich	71463
20% SDS	Sigma Aldrich	5030
3M Sodium Acetate (pH5.2)	Sigma Aldrich	S7899
5M NaCl	Sigma Aldrich	S5150
Ethanol	Sigma Aldrich	E7023
Guanidine hydrochloride	Sigma Aldrich	G3272
Isopropanol	Sigma Aldrich	650447
PEG-8000	Sigma Aldrich	89510
Proteinase K	Sigma Aldrich	P6556
Tween-20	Sigma Aldrich	P9416
Water	Sigma Aldrich	W4502
10X Buffer Tango	Thermo Fisher Scientific	BY5
50X Denhardt’s solution	Thermo Fisher Scientific	750018
AccuPrime Pfx Polymerase (2.5U/μL)	Thermo Fisher Scientific	12344032
ATP	Thermo Fisher Scientific	R0441
dNTP Mix	Thermo Fisher Scientific	R1121
Dyna MyOne Streptavidin C1 beads	Thermo Fisher Scientific	65002
FastAP (1U/μL)	Thermo Fisher Scientific	EF0651
GeneAmp 10X PCR Gold Buffer	Thermo Fisher Scientific	4379874
Human Cot-I DNA	Thermo Fisher Scientific	15279011
Klenow Fragment (10U/μL)	Thermo Fisher Scientific	EP0052
Maxima Probe qPCR 2xMM	Thermo Fisher Scientific	K0233
Maxima SYBR Green kit	Thermo Fisher Scientific	K0251
Maxima SYBR Green kit	Thermo Fisher Scientific	K0253
Salmon sperm DNA	Thermo Fisher Scientific	15632–011
SSC Buffer (20X)	Thermo Fisher Scientific	AM9770
T4 DNA Ligase	Thermo Fisher Scientific	EL0012
T4 DNA Ligase, HC (30U/μL)	Thermo Fisher Scientific	EL0013
T4 DNA Polymerase	Thermo Fisher Scientific	EP0062
T4 Polynucleotide Kinase	Thermo Fisher Scientific	EK0032
Acetic Acid,Glacial (TraceMetal Grade)	Fisher Chemical	Cat# A507-P212
7M HNO_3_ (Optima)	Fisher Chemical	Cat# A467–2
6M HCl (TraceMetal Grade)	Fisher Chemical	Cat# A508–4
0.05M HNO_3_ (Optima)	Fisher Chemical	Cat# A467–2
2% Sodium Hypochlorite Solution	Millipore Sigma	Cat# XX0637–76
30% H_2_O_2_ (GR ACS Grade)	Millipore Sigma	Cat# HX0635–2
0.1M CH_3_COOH (GR ACS Grade)	Millipore Sigma	Cat# AX0073–6

## INCLUSION AND DIVERSITY

We support inclusive, diverse, and equitable conduct of research.
