# Supplementary material for: Papuan Admixture Predated the Settlement of Palau
Source: Cell. Author manuscript; Available in PMC 2026 Mar 28. (PMC13025617; doi:10.1016/j.cell.2026.02.011)
Supplement: DocumentS1_Supplemental_Information — Document S1. Archaeological context, Tables S1 and S3, and supplemental references [file NIHMS2150805-supplement-DocumentS1_Supplemental_Information.pdf]

# DOCUMENT S1 - SUPPLEMENTAL INFORMATION

## Papuan Admixture Predated the Settlement of Palau

Yue-Chen Liu, Joanne Eakin, Jolie Liston, Rosalind Hunter-Anderson, Calvin Emesiochel, Kiblas Soaladaob, Sunny O. Ngirmang, Olivia Cheronet, Carla S. Hadden, Alexander Cherkinsky, Matthew Spriggs, Keith Prufer, Swapan Mallick, Nadin Rohland, Ron Pinhasi, David Reich

**This PDF file includes:**

Archaeological context, burial practices, and site descriptions

Tables S1 and S3

Supplemental references

**Additional supplementary materials are provided in separate spreadsheets, including:**

Tables S2 and S4

## Archaeological Context, Burial Practices, and Site Descriptions

Current archaeological data indicate Palau's Rock Islands and Babeldaob first supported human visitors around 3200-3000 BP[20,57–59]. The earliest securely dated archaeological deposits on Palau come from the Rock Islands, in a burial cave at Chelechol ra Orrak[60], a temporary occupation site on Ulong's back beach[20], and Ucheliungs cave[58]. With Palau's earliest dates derived from marine shell and human bone with a substantial marine dietary protein contribution, radiocarbon calibrations are dependent on locality-specific marine reservoir correction values, which produce variable results that differ by hundreds of years. Palau's $\Delta R$ values for the lagoon and the open ocean range from 168±43 to -250±50, with a $\Delta R$ value of -140±35 for the Marine20 calibration curve[21–23,58,61,62].

Palau's pre-European Contact cultural sequence may be divided into four loosely bounded major periods: Colonization c. 3200-2800 BP, Expansion Period c. 2800-2400 BP, Earthwork Period c. 2400-1100 BP, and Stonework Period c. 1100 BP to European contact in 1783[33]. These eras are based on changing land use and associated transformations in architecture, artifacts, and other cultural practices. The main body of islands in the Palau archipelago developed together through a robust trade and exchange network as evidenced by oral traditions pertaining to the Stonework Period, including place names, complementary artifact and midden (food and other debris) assemblages, and similar monumental stonework architecture.

The Colonization Period is characterized by marine foraging and intermittent use of small camps on the Rock Islands where a variety of marine species were collected[20,63]. Some of the human interments in the cemetery at the Rock Island rock shelter of Chelechol ra Orrak date to this period. With relatively substantial tectonic subsidence, a fluctuating sea level, and erosional deposits destroying or heavily altering evidence of early settlement along Babeldaob's coastal margins, the earliest known cultural deposits, dating to the latter part of this period, are currently found only in the interior uplands of this large volcanic island[59,64,65].

During the Expansion Period, the number and kind of settlements increased throughout the archipelago. On Babeldaob, construction of terraces occurred behind the coastal lowlands possibly to form level land for habitation and agriculture in steeply sloped terrain. The human

activities in Babeldaob interior likely included foraging, surveillance, and, where possible, habitation and horticulture[64]. Coastal subsistence strategies focused largely on collecting nearshore mollusk and fish species and likely horticulture in available wetlands[20,66,67]. As in the remainder of Micronesia, Palau's flora derives largely from the East-Asian and Indo-Malayan region[68]. With the poor preservation of botanical remains in Babeldaob's strongly acidic soils, there is currently very limited direct evidence for the earliest cultigens. Native edible plants available to the colonizers first alighting on Palau's shores include coconut (*Cocos nucifera*), giant swamp taro (*Cyrtosperma merkusii*), wild native breadfruit (*Artocarpus mariannensis*), pandanus (*Pandanus tectorius*), the tropical almond (*Terminalia catappa*), and the Tahitian chestnut (*Inocarpus fagifer*)[69–71]. Edible varieties of wild yam (*Dioscorea* spp.) were likely present before colonization. The most significant early introduced food crop, and a dominant staple since colonization, is the true taro (*Colocasia esculenta*) already domesticated in the Indo-Malayan region. Species of edible banana (*Musa* spp.) and domesticated breadfruit (*Artocarpus altilis*) likely originated in New Guinea[72,73], before being introduced by Palau's early settlers. At present, the timing of the arrival of early introduced food crops in Palau remains uncertain within the archaeological sequence. Consequently, both possibilities remain open: they may have arrived either with the earliest migrants or with later human movements.

The Rock Islands were used for resource procurement, small-scale temporary habitation, and burials[20,22,60,63]. The oldest cultural deposits on Ulong Island contain abundant marine shell and fish bone, and earthenware fragments tempered with volcanic sand or a hybrid calcareous and terrigenous sand. These ceramics occasionally display decoration, including a few with red slip, linear impressions and incising, some possibly with lime-infilling[20]. The earliest vessel forms, unearthed in the Rock Islands and interior Babeldaob, include simple and necked globular bowls and ovoid jars with everted rims[74–76].

Rock painting on highly visible fissured cliffs and marine notches on eight Rock Islands may have begun during the Expansion Period[32]. The paintings are executed in red pigments and vary in size from several centimeters to a meter tall. Stylized, abstract, geometric, and linear motifs dominate the painting corpus while anthropomorphic, zoomorphic, and other figurative forms occur less frequently. Apparent stylistic, locational, and chromatic resemblance to rock art in eastern Indonesia and West Papua, which dates to around 3500 BP, suggests that knowledge

of painting may have been brought to Palau by the early settlers. However, none of these images have been directly dated.

In the Earthwork Period, elaborate and diverse earthen forms were constructed on Babeldaob. These features cover about 20% of the island's land surface[64]. Forming a vast engineered landscape, the enormous earthworks supported habitation areas and agricultural production systems. They were used for water management, trails, and meeting sites; they served as boundary markers, barriers, smoke signal towers and other defensive functions; and they played ceremonial and ritual roles. In addition to these practical purposes, the distributional patterning, size, and morphology of the earthwork complexes indicate that as a whole, they served as multi-faceted symbols of political power[64,77]. Palau's earthworks are the oldest large-scale monumental architecture in the Pacific. Paleoenvironmental data suggest permanent settlements in the Rock Islands may have been established close to 2000 BP[78] with archaeological evidence indicating permanent settlement by c. 1700 BP[22].

Undecorated grog-tempered vessels replaced the earlier sand-tempered ceramics[20,60]. The variety of vessel forms expanded to include unrestricted, shallow oval dishes or plates and simple bowls with thickened and straight-sided rims. Around 1700 BP, there is no evidence of fabrication of globular bowls and everted rims, and incurving rim forms decreased in abundance as well. Bowls with flanged rims appear by 1400 BP, after which they become progressively more common. Radiometric dates indicate Palauan pots were painted by at least 1800 BP and that this form of surface decoration continued until European contact[76]. Petrographic analysis indicates pottery wares originated in Palau's volcanic islands and were exchanged throughout the archipelago's limestone islands, which lacked clay-bearing soils. Palau ceramics have been observed as far afield as the atoll of Ngulu and Fais Island in Yap[60,79].

The Stonework Period began during a poorly documented transitional phase of variable settlement patterns and subsistence strategies, perhaps a result of a combination of climate warming and sociopolitical upheaval[33,64] This culminated in the establishment of coastal-based clusters of nucleated villages characterized by massive and elaborate stone architecture (stone burial platforms, paths, bathing pools, pavings, and docks) and a subsistence economy based on wetland cultivation of *kukau* (true taro, *Colocasia esculenta*), fruit and nut trees such as

breadfruit and Tahitian chestnut, and marine resources. Village architecture on the Rock Islands was based on limestone blocks, while on the volcanic islands, structures were made of local basalt. Oral histories and ethnohistoric accounts tell of a hierarchically structured society guided by customary forms of institutionalized competition and reciprocity.

Earthenware containers during this time were typically deep or restricted jars and round and oval, open bowls, dishes, and plates with thickened, flanged, or straight-sided rims. Decorated vessels, whether painted, incised, or impressed, were progressively less common.

A long period of drier climate beginning c. 600 BP, identified in paleo-precipitation studies[80,81], dramatically reduced the supply of potable water in the Rock Islands, leading to oral histories of resource deprivation and social conflict. By 500 to 300 BP the Rock Island stonework settlements were abandoned as villagers migrated to the volcanic islands with secure water sources[63].

**Palauan Burial Practices**

Wide variation in Palau's pre-European Contact burial practices resulted from a combination of geological necessity and temporal variations in Palauan cultural practices[82–84]. The earliest Palauan human interments originate from Rock Island caves and rock shelters. Any early burials that may have occurred on the coastal margins of volcanic islands and atolls have been destroyed through tectonic subsidence, a fluctuating sea level, and sedimentation[20,63]. Pre-European Contact burials have been found in Babeldaob's earthworks and in stone platforms dating to the Stonework Period throughout the archipelago. Continuity in mortuary ritual is seen in the placement of red clay under the deceased or in the staining of the body or burial mat with a red substance, identified in burials in limestone caves, earthworks, and stone platforms, extending from at least 2000 BP to very recently.

Eleven Rock Island caves and rock shelters containing human skeletal remains are documented, with many more known to the local population. Despite the use of limestone caves for interment of the dead, there are no oral traditions directly linked to cave burials other than a few places mentioned in passing as old graveyards and two identified as burial places of a high chief. Four burial caves have been archaeologically investigated to some degree: the cemetery at Chelechol ra Orrak, and the three included in this study, Ngermereues (Koror), Omedokl, and Ucheliungs.

The Chelechol ra Orrak rock shelter on the Rock Island of Orrak, a kilometer east of southeastern Babeldaob, contains one of the earliest known cemeteries (n=55 individuals) in Remote Oceania[18,60,85]. Initial burial activity began between 2990-2740 calBP and continued until ~1600 BP, about 1300 years[57]. Calibrated radiocarbon dates show a similar 1400-year span of interment at the burial caves at Ucheliungs (beginning ~2700 BP), Ngermereues Ridge (~2400 BP), and Omedokl (~1700 BP), with interment in the latter not ceasing until about 700 BP. Whether the caves were used for human burial continuously or intermittently is unknown, in part because radiocarbon dates have been obtained on only a few of the interred individuals.

Bone preservation in the caves is generally fair, ranging from extremely friable and weathered to fossilized elements. Except at Chelechol ra Orrak rock shelter where burials, some articulated, descend deep into beach sand[82], most cave skeletal elements are in secondary contexts, disarticulated, highly fragmented, and dispersed across cave surfaces, below loose rubble or thin layers of sediment, and in dense concentrations on shelves along the sides of chambers. This suggests many individuals were originally placed on the surface of the cave floor or in shallow subsurface graves[58] then secondarily redeposited due to tidal surges, cave collapse, bioturbation, and other natural mechanisms. The burials are unmarked, with no archaeological features indicating their presence.

A broad demographic profile of males and females, ranging in age from fetal to older adults, are represented in the limestone cave burial populations. The diversity of individuals indicates no age or sex preference of burial practices. Specific burial caves may have been restricted to members of a specific clan or lineage, to high-status individuals, or to certain occupations, but further research is needed to clarify this.

Limestone cave interments are occasionally accompanied by a diverse collection of grave goods including shark teeth (some drilled), unworked marine shells, earthenware pottery vessels, some decorated with red paint, a bird bone awl/pick, a piece of carved bone (potentially part of a harpoon), marine shell decorative ornaments, pearl shell scrapers (*chesiuch*), stone and shell adzes, and painted basalt stones. Grave goods indicate frequent interaction and exchange between the volcanic and limestone islands of the archipelago, as the latter lacked clay or

appropriate stone for tools. The diversity and uneven distribution of these goods suggest a degree of social, sex, or age differentiation. According to ethnohistoric and contemporary Palauan customs, pearl shell tools, used for grating taro and coconut, would be interred with women while shark teeth may have been male goods.

Evidence of ritual activity is a red matrix that underlies some limestone cave and earthwork burials[64,84]. The body may have been placed on a thin layer of naturally red volcanic sediment transported to the gravesite for the burial ceremony. Alternatively, the red matrix could be staining from a dyed mat wrapped around or under the body or from a substance, such as turmeric (*reng*), applied to the body during or prior to burial. The ritual application of "paint" on the deceased's face and chest during burial preparations is noted in ethnographic accounts from 1876 with the practice continuing until recently.

Cave sites also contain varying degrees of non-human faunal material, generally unworked marine shell from the adjacent environments of mangrove mudflats, lagoon shelves, seaward reef slopes, or beach rock deposits, but also sea urchin, fish, and bird bone[18,66]. These remains are likely related to a combination of subsistence activities, burial offerings, and post-interment deposition during king tides and storm surges.

The absence of archaeological features and domestic cultural debris suggests that some limestone caves were used exclusively for mortuary activities; however, given their long period of use, cave functions may have changed through time. Radiocarbon assays and artifact and faunal assemblages suggest Ucheliungs supported small-scale temporary habitation before and after its use for human interments[58]. Given the devastating impact of the infrequent typhoons on the islands' inhabitants, it is probable those caves not prone to flooding also served as storm shelters. Occupation of limestone caves as a refuge and burial place by those defeated in battle is related in an oral tradition, one of the few that states that caves were used for burial purposes[84].

Babeldaob's strongly acidic soils rapidly disintegrate skeletal elements and remains are infrequently encountered in Earthwork Period sites there. The radiocarbon date of 1469-1248 calBP, from one of the Ngkeklau remains analyzed in this study, places the Babeldaob burial in this era. Although the precise grave location is unknown, the skeletal elements association with

fragments of limestone and coral indicates it originated from coastal beach or cave deposits. Surface indicators of upland grave sites are poorly defined. Interior interments are archaeologically identified by burial pits and the remains of funerary activities such as capping clay layers, sea sponge mats, a red matrix, small basalt paving, rough alignments or stone-faced pits, and occasionally burial goods, most often complete pottery vessels.

The practice of earthworks serving mortuary functions began by at least 2000 BP as evidenced by the structured communal burial sets unearthed on low step-terraces next to small-scale habitation areas on Babeldaob[83]. By about 1300 BP, burials accompanied by grave goods and ritual activity are found on the summits of the steep-sided, flat-topped high points called crown earthworks. Placement of individuals in such highly visible and spatially restricted spaces may have been done to affirm the deceased kin group's power and prestige. These temporal transformations in burial practices are proposed to identify a social shift from corporate strategies of group integration to individual distinctions in rank and a hierarchical social structure[83].

In the Stonework Period, Palauans buried their dead in simple earth pits in the *odesongel* (burial platform) of the lineage or clan of the deceased, in stonework villages throughout the archipelago. Some clan members continue the tradition, although it is currently more common to place the dead in community cemeteries. The associated house stood on posts adjacent to the length of the *odesongel*. Those from across the sea are buried in coral crypts in the *odesongel*. In some instances, piles of stone or stone slabs serve as burial markers (*bluks*). The fear of spirits of the deceased resulted in those who had a "strange" death—those beheaded in war, killed by marital revenge, women who died in childbirth, and suicides—to be buried without honors outside clan land[84]. It is unknown where these interments occurred. Some burials, said to be related to battles, are found along the coastal plains seaward of the stonework villages.

Grave goods dating to the Stonework Period include jewelry composed of small glass, and occasionally, shell, beads, burial mats (one identified as of charred breadfruit [*Artocarpus sp.*] leaves), and the occasional continued association with red or red-stained clay. There is very limited osteological data on Stonework Period interments due to the paucity of non-disintegrated skeletal material and the very few *odesongel* that have been archaeologically documented.

## Site Descriptions

### Ngermereues Ridge Burial Cave (Site No. OR-3:30), Koror Island

Located at the northeastern tip of the mixed volcanic and limestone island of Koror, just south of the channel separating it from the volcanic island of Babeldaob, Ngermereues Ridge is a narrow, southerly trending escarpment bordered on the east and west by mangrove forest. This ridge and its neighboring ridges to the south are collectively referred to as “Euatel Ngermid”—fortification for Ngermid, a village just to the south, as they served as a natural fortification to ward off war parties approaching from the north and protect the village from typhoons[84]. Similarly, the ridge holds a World War II Japanese defensive complex composed of stacked limestone and concrete enclosures, bunkers, walls, and other fortifications.

During archaeological data recovery prior to quarrying activities which leveled much of the ridge, Rieth and Liston (2001)[84] documented 36 traditional Palauan burials in two adjacent limestone solution caves at the south end of the ridge. Human remains were recovered throughout the 12 chambers within the ~120 square meter area of the Feature 1 cave and the single-chambered ~60 square meter Feature 2 cave. Flowstone, pillars, and other mineral formations are abundant in the chambers and the large amounts of rubble covering the cave’s floor have crushed many of the skeletal elements.

Some of the interments are associated with burial goods, including shark teeth, unworked marine shell, traditional earthenware pottery sherds (two of which are painted), a bird bone awl/pick, a shell pendant, a piece of lithic shatter, decorative cowry shell artifacts, and pearl shell scrapers. The caves also contained some historic artifacts associated with Japanese World War II activities. Three burials were accompanied by a thin bed of distinctive red clay, an indication of an associated burial ceremony.

The highly fragmented, displaced, and friable human remains were scattered on the surface, below loose rubble, and in dense concentrations on shelves along the sides of the chambers. The minimum number of individuals (MNI) removed from Feature 1 is 32 and Feature 2 had an MNI of four. Of these, 16 are adults (>19 years), two are probable adults, six are subadults with at least two individuals <8 years of age, with the age of the remaining 12 individuals

indeterminate. Only three individuals were complete enough for sex determination, and all are probably female.

Stone et al. (2019)[67] performed stable isotope analysis on three Ngermereues adults of undetermined sex and produced a mean bone collagen value for $\delta^{13}C$ of -14.8‰ and $\delta^{15}N$ of 11.4‰, a mean bone apatite value of $\delta^{13}C$ of -7.3‰, and a $\delta^{13}C_{ap\text{-}co}$ spacing averaging 7.5‰. The results reflect a primarily marine protein-based diet supplemented with a variety of terrestrial plants. Consumption of sugarcane or marine plants like kelp or seaweed or other $^{13}C$-enriched source of carbohydrate is suggested by the elevated $\delta^{13}C$ values for bone apatite. The three bone samples from Ngermereues Ridge were calibrated with a 50 percent mixed marine/terrestrial curve and a $\Delta R = -140 \pm 35$. The earliest date range was from a left tibia in Feature 2 cave which produced a calibrated date range of 2498-2140 calBP (CAMS-65957); the two samples from Feature 1 rendered radiocarbon dates of 1551-1294 calBP (CAMS-65958) and 1251-925 calBP (CAMS-65956) (see **Figure S1** and **Table S3.2** for details). In the present study, we generated three additional radiocarbon dates on samples also analyzed for ancient DNA, which we calibrated to 1691-1398 calBP (PSUAMS-177530, 1695-1414 calBP (UGAMS-71517), and 1588-1352 calBP (UGAMS-74376). Taken together, these six dates indicate that the caves were used for burying the deceased over a period of at least 900 years. Whether the caves were used continuously or intermittently is unknown although the gaps are probably a result of acquiring radiocarbon dates on only five of the at least 32 individuals interred there.

The Ngermereues Ridge burials include individuals representing a range of ages from infants to older adults, with females and likely males present. The diversity and distribution of grave goods suggest a degree of social, sex, or age differentiation. Based on the evidence from the bones and grave goods, the population chewed betel nut, consumed a variety of marine and terrestrial resources, and engaged in physical activities (e.g., fishing, hunting, gardening) which led over the normal course of life to osteoarthritis in some individuals.

**Omedokl Burial Cave (Site No. OR-15:8)**

Human burials were found in the cave which forms the entire interior of the Rock Island of Eil ra Rechiklau/Ii ra Omedokl (Site No. OR-15:8). Omedokl, only ~1 ha in size, lies just off the south coast of the large Rock Island of Ngeruktabel. According to traditional history, the cave served

as the burial place for the children of the Rechiklau, a Rock Island chief (Osborne 1966:436). Concentrated areas of fragmented human remains were densely scattered across the surface and to a depth of at least 40 cm and several relatively complete crania were heavily embedded in calcium carbonate flowstone.

The 5 m wide and 2 to 3 m high north-facing cave entrance, near the high tide mark, leads down a 45 m long southwest-oriented passage into a 20 m wide and 46 m long chamber. The far north end of the chamber contains sand deposits backed by flowstone. The cave surface is relatively level and covered in calcareous sediments, some limestone rubble, and occasionally, massive boulders resulting from roof fall.

Osborne (1966)[86] found burial goods in the cave including a traditional earthenware plate and was told of a Japanese finding a painted pottery bowl and lid containing stone adzes and shell items. Pot hunters have removed the additional traditional earthenware vessels and other artifacts reported to have once been in the cave. Currently, some pottery sherds and marine shell fragments are strewn across the surface.

Berger et al. (2008)[87] collected 110 bone fragments, including 36 teeth, from the surface of Omedokl. The remains were in disturbed contexts, likely secondarily redeposited by wave action during strong storms and bioturbation. Berger et al. (2008)[87] focused on the specimens' dimensions and provided very little information on age, sex, pathologies, and the like. They identified at least one male and one female and at least one juvenile among the primarily adult assemblage.

Three published radiocarbon assays on the Omedokl bone specimens produced calibrated date ranges (50/50 percent marine-terrestrial with a $\Delta R$=-140±35) of 784-552 calBP (B:OR-15:18-102), 1350-1075 calBP (B:OR-15:18-100), and 1895-1567 calBP (B:OR-15:18-101) (see **Figure S1** and **Table S3.2** for details). For this study, we newly generated three dates calibrated to 1501-1270 calBP (PSUAMS-17752), 1591-1350 calBP (UGAMS-74371), and 723-548 calBP (UGAMS-74372) Omedokl was thus used for human interments for more than a millennium although it is unknown whether burials were intermittent.

**Ucheliungs Burial Cave (Site No. OR-14:8)**

Surface collections of the large quantity of mineralized, mostly disarticulated, and highly fragmented human skeletal elements covering the floor of the Ucheliungs Rock Island burial cave (Site No. OR-14:8) and small-scale excavation recovered over 2000 generally isolated human bone specimens and up to 44 teeth[18,58,87,88]. At least one relatively complete cranium remains heavily embedded in calcium carbonate flowstone.

Ucheliungs is a small (c. 2 ha) Rock Island located about 3 km east-southeast of Koror Island, in a narrow channel between the large Rock Islands of Ngermeuangel and Ulebsechel. Ucheliungs' ~130 square meter main cavern opens to the ocean on the east with a small, narrow chamber extending some 10 m towards the north off of the main cavern. Rubble is scattered across the floor with large limestone blocks piled in the center of the main cavern, a result of a collapsed roof. An up to 50 cm thick deposit of calcareous sand caps the underlying flowstone. The highly disarticulated and fragmentary nature of the skeletal material suggests their secondary deposition possibly from bioturbation, roof collapse, and wave action during typhoons and king tides.

Human remains were densely scattered across the cave floor, along with limited artifacts (generally earthenware sherds) or non-human faunal material. While one of the two test units placed in the main chamber produced no non-human faunal remains and only a few fragments of pottery[88], the other 1 $m^2$ test unit unearthed 30 small, highly weathered, volcanic sand tempered earthenware sherds, two shark teeth, a fish vertebra, a bird coracoid, land snails, sea urchin, barnacles, and abundant crab remains[18,58]. Also present were 55 taxa of mineralized marine mollusks most originating from the adjacent shallow reef environments. No single marine shell taxon is dominant.

Although no discrete individuals or articulated burials were identified, Stone et al. (2017)[58] provide a rough estimate of MNI, based on nonrepetitive elements, of at least six individuals—three adults and three juveniles, ranging in age from infancy to adulthood. The age of individuals[87] in the assemblage varied from neonate to juvenile, sub-adult, and adult[88]. The specimens were too fragmented to accurately determine sex.

Ucheliungs has previously produced twelve radiocarbon assays: eight on human bone[87] and four on marine shell used for subsistence[58]. The bone assays, of unknown provenience, range from 2699-1097 calBP, calibrated as 50/50 percent marine-terrestrial with a ΔR=-140±35, while the three accepted marine shell dates, from a test unit, range from 3485-2611 calBP, and one marine shell date, probably an outlier, range from 4160-3762 calBP (Marine20 Curve at ΔR=-140±35 years) (see **Figure S1** and **Table S3.2** for details). These dates are consistent with the three newly reported dates on bone we report here for samples with DNA: 2924-2710 calBP (PSUAMS-17751), 2776-2495 calBP (PSUAMS-17755), and 1508-1288 calBP (PSUAMS-17754). In combination, the date ranges suggest Ucheliungs was used for human interments over a period of ~1500 years beginning about 2800 years ago, and that the cave may have been used for other activities prior to the placement of burials.

The presence of a diverse individuals, ranging in age from infancy to adulthood, indicates there were no age restrictions for those interred in Ucheliungs[58]. The artifact and faunal assemblage as well as the radiocarbon assays suggest Ucheliungs supported small-scale temporary habitation before and after the deposition of human remains.

**Ngkeklau, Ngaraard**

Specimens were collected from a skeletal assemblage stored at the Belau National Museum for this study. The remains were collected in 1991 with an inventory conducted at the Bishop Museum in Honolulu, Hawaii identifying at least five individuals: four adults and one child[89]. Institutional memory at the Belau National Museum places the bones in the Ngkeklau region of Ngaraard state in northeastern Babeldaob Island (thought to be most likely) or from Ngerkeklau, a c. 8 ha a mixed volcanic and limestone island adjacent to the northern end of Babeldaob in Ngerchelong state. Ngerkeklau, currently uninhabited, once supported a stonework settlement. Both locations could have provided the coral and limestone fragments associated with the stored remains.

Ngkeklau, one of five autonomous village areas of Ngaraard, is at the southern base of the state on the east coast. Although the village area encompasses about 1300 ha, only the traditional stonework village of Ngkeklau has been occupied for the past 80 years. A few scattered homesteads line the coastline behind wide expanses of mangrove forest. The human remains

may have been collected during various road construction or other infrastructure efforts in Ngkeklau village or from the dirt tracks leading from the village, although there were no associated archaeological investigations to confirm this. The remainder of the village area contains extensive pre-European Contact earthworks, abandoned stonework villages, stone paths, and taro pond fields.

Currently, Ngkeklau villagers live along a strip of gently sloping land by the Japanese era concrete road that parallels a narrow sandy coastal plain fronting the shallow lagoon. Behind, west of the flat lands, the terrain rises into a southeasterly trending ridge that supports the unoccupied stonework village. The stone platforms and paths composing the traditional village are built onto low step-terraces shaped into the ridge perhaps thousands of years ago that are now covered in jungle vegetation and economically useful plants.

Ngkeklau, archaic name Ngotel, is said to have been settled by migrants from Klouloikull and Medal in Oikull, Ngeruangel Rock Island, and by Yap islanders. Some local people say the name Ngkeklau derived from the place in Yap named Keslau. There are no radiocarbon assays from Ngkeklau village, but Ngaraard state has produced over 70 earthwork radiocarbon assays with dates extending back to 2700 BP[33].

## Table S1 Archaeological periods in Palau

| Period | Years BP | Description |
|---|---|---|
| Colonization | 3200-2800 | Characterized by marine foraging and intermittent use of small camps on the Rock Islands where a variety of marine species were collected[20,57,59,63]. On Babeldaob's coastal margins, the earliest sites may have been largely obliterated over time by substantial tectonic subsidence, declining sea level and eroded upland deposits. Some human burials from the Rock Island shelter of Chelechol ra Orrak date to this period[57]. |
| Expansion | 2800-2400 | The number and kind of sites increased throughout the archipelago. On Babeldaob, terraces were constructed in steeply sloped terrain for habitation and agriculture. The Rock Islands were used for resource procurement, temporary occupation, and human interment[20,22,60,63]. Painting images on highly visible fissured cliffs and marine notches at six Rock Islands may have begun at this time, perhaps signifying ownership claims[32]. |
| Earthwork | 2400-1100 | Elaborate and diverse earth architecture was constructed on Babeldaob, covering about 20% of the island's surface area[64]. Enormous earthworks supported most habitations, cultivation, defensive elements, infrastructure such as water management, and ceremonial activities including burials. Small settlements in the Rock islands may have been established close to 2000 BP[78] but archaeological evidence indicates permanent settlement by c. 1700 BP[22,57]. |
| Stonework | 1100-European contact | This was a period of variable settlement patterns and subsistence strategies, perhaps caused by a major climatic oscillation and sociopolitical upheaval[33,64] that culminated in the establishment of coastal-based clusters of nucleated villages of massive and elaborate stone architecture and a highly competitive hierarchical socio-political system. A long period of drier climate[80,81] and more frequent El Nino droughts dramatically reduced the supply of potable water in the Rock Islands and is reflected in oral histories of resource deprivation and social conflict. By 500 to 300 BP, the Rock Island stonework settlements were abandoned as villagers migrated to the volcanic islands with secure water sources[63]. |

**Table S3.1 New radiocarbon dates reported in this study**

| ID | Locality | Material | Identifier | $\delta^{13}C$ (‰) | $\delta^{15}N$ (‰) | C:N Atomic | $^{14}C$ age (BP) | 95% CI of calibrated dates (calBP) 0% | 20% | 50% | 100% |
|---|---|---|---|---|---|---|---|---|---|---|---|
| I41048 | Ucheliungs | Ultrafiltered | PSUAMS-17751 | -13.18 | 13.01 | 3.28 | 2860±30 | 3071-2876 | 3001-2789 | 2924-2710 | 2765-2403 |
| I41049 | Ucheliungs | XAD amino acids | PSUAMS-17755 | -13.75 | 9.80 | 3.12 | 2725±30 | 2871-2760 | 2850-2736 | 2776-2495 | 2668-2276 |
| I41056 | Ucheliungs | Ultrafiltered | PSUAMS-17754 | -15.44 | 12.04 | 3.29 | 1670±25 | 1692-1517 | 1550-1403 | 1508-1288 | 1345-1050 |
| I42651 | Koror Quarry | Ultrafiltered | PSUAMS-17753 | -14.97 | 11.32 | 3.28 | 1815±20 | 1780-1624 | 1717-1579 | 1691-1398 | 1505-1200 |
| I42654 | Koror Quarry | Non-Ultrafiltered | UGAMS-71517 | -15.73 | 11.20 | 3.26 | 1840±25 | 1825-1646 | 1745-1594 | 1695-1414 | 1527-1235 |
| I42656 | Koror Quarry | Non-Ultrafiltered | UGAMS-74376 | -15.42 | 11.29 | 3.23 | 1760±20 | 1712-1599 | 1697-1532 | 1588-1352 | 1440-1138 |
| I41042 | Omedokl | Non-Ultrafiltered | UGAMS-74371 | -13.99 | 11.67 | 3.26 | 1760±25 | 1714-1575 | 1699-1530 | 1591-1350 | 1446-1134 |
| I41043 | Omedokl | Ultrafiltered | PSUAMS-17752 | -13.91 | 11.45 | 3.29 | 1650±25 | 1687-1415 | 1536-1400 | 1501-1270 | 1329-1027 |
| I41045 | Omedokl | Non-Ultrafiltered | UGAMS-74372 | -15.34 | 11.93 | 3.25 | 890±20 | 903-731 | 780-680 | 723-548 | 604-317 |
| I41051 | Ngkeklau | Non-Ultrafiltered | UGAMS-71516 | -16.15 | 10.97 | 3.29 | 1630±20 | 1545-1413 | 1526-1386 | 1472-1248 | 1300-1003 |
| I41052 | Ngkeklau | Non-Ultrafiltered | UGAMS-74374 | -15.31 | 11.63 | 3.26 | 1760±20 | 1712-1599 | 1697-1532 | 1588-1351 | 1441-1135 |
| I41048 | Ucheliungs | Non-Ultrafiltered | UGAMS-71515 | -11.69 | 12.83 | 3.32 | 2720±20 | Non-ultrafiltered and inconsistent date | | | |
| I41049 | Ucheliungs | Non-Ultrafiltered | UGAMS-74373 | -15.50 | 12.50 | 3.60 | 3280±25 | C:N ratio out of the recommended range of 2.9-3.4 | | | |
| I41056 | Ucheliungs | Non-Ultrafiltered | UGAMS-74375 | -15.47 | 11.51 | 3.30 | 1720±20 | Non-ultrafiltered and consistent date | | | |
| I43894 | Koror Quarry | Non-Ultrafiltered | UGAMS-74377 | -24.82 | n/a | n/a | 4610±50 | An extreme outlier of $\delta^{13}C$‰ and no $\delta^{15}N$‰ | | | |
| I43044 | Koror Quarry | Non-Ultrafiltered | UGAMS-74378 | -17.56 | 11.70 | 3.68 | 1180±25 | C:N ratio out of the recommended range of 2.9-3.4 | | | |
| I41043 | Omedokl | Non-Ultrafiltered | UGAMS-71514 | -13.50 | 12.10 | 3.38 | 1700±20 | Non-ultrafiltered and consistent date | | | |

**Notes:** Calibrated dates combine IntCal20 and Marine20 assuming 0%, 20%, 50%, and 100% marine dietary protein with ±10% variance. ΔR = -140 ± 35 $^{14}C$ years[21] is applied for Palau. The results presented in the main text are based on calibrations assuming 50% marine dietary protein. We do not use the six dates below the line for the reasons specified.

## Table S3.2 Published dates in Ucheliungs Cave, Koror Quarry, and Omedokl Cave

| Identifier | Locality | Material | Specimen Type | δ13C (‰) | δ15N (‰) | $^{14}C$ age (BP) | ± | Calibrated 95.4% CI Upper | Calibrated 95.4% CI Lower | Reference |
|---|---|---|---|---|---|---|---|---|---|---|
| B:OR-14:8-1200 | Ucheliungs | collagen | human bone | -15.10 | 11.40 | 2550 | 50 | 2699 | 2211 | [87] |
| B:OR-14:8-1201 | Ucheliungs | collagen | human bone | -15.30 | 11.40 | 2530 | 50 | 2681 | 2181 | |
| B:OR-14:8-1202 | Ucheliungs | collagen | human bone | -15.00 | 11.50 | 2280 | 50 | 2306 | 1899 | |
| B:OR-14:8-1203 | Ucheliungs | collagen | human bone | -14.70 | 11.80 | 2260 | 50 | 2300 | 1880 | |
| B:OR-14:8-1204 | Ucheliungs | collagen | human bone | -15.90 | 13.00 | 2190 | 50 | 2151 | 1773 | |
| B:OR-14:8-1205 | Ucheliungs | collagen | human bone | -14.40 | 12.60 | 2400 | 40 | 2365 | 2045 | |
| B:OR-14:8-1206 | Ucheliungs | collagen | human bone | -17.00 | 13.00 | 1520 | 40 | 1370 | 1097 | |
| B:OR-14:8-1207 | Ucheliungs | collagen | human bone | -14.00 | 13.00 | 1570 | 40 | 1406 | 1151 | |
| D-AMS 017432 | Ucheliungs | unknown | Marine shell (*Cypraea tigris*) | n/a | n/a | 3000 | 30 | 2951 | 2611 | [58] |
| D-AMS 016831 | Ucheliungs | unknown | Marine shell (bivalve, likely *Cardiidae sp.*) | -17.60 | n/a | 3450 | 30 | 3485 | 3142 | |
| D-AMS 017433 | Ucheliungs | unknown | Marine shell (*Fimbria sp.*) | n/a | n/a | 3370 | 30 | 3398 | 3038 | |
| D-AMS 017434 | Ucheliungs | unknown | Marine shell (bivalve, likely *Cardiidae* sp.) | n/a | n/a | 3960 | 30 | 4160 | 3762 | |
| CAMS-65956 | Koror Quarry | collagen | human bone | -14.70 | 11.20 | 1350 | 40 | 1251 | 925 | [67,84] |
| CAMS-65957 | Koror Quarry | collagen | human bone | n/a | n/a | 2480 | 40 | 2498 | 2140 | |
| CAMS-65958 | Koror Quarry | collagen | human bone | n/a | n/a | 1720 | 40 | 1551 | 1294 | |
| B:OR-15:18-100 | Omedokl | collagen | human bone | -17.00 | 10.30 | 1500 | 40 | 1350 | 1075 | [87] |
| B:OR-15:18-101 | Omedokl | collagen | human bone | -16.50 | 9.20 | 2000 | 40 | 1895 | 1567 | |
| B:OR-15:18-102 | Omedokl | collagen | human bone | -14.60 | 13.00 | 940 | 40 | 784 | 552 | |

Uncalibrated direct radiocarbon dates and isotopic data were obtained from the literature. For human bones, we calibrate these dates based on a mixed calibration curve combing 50% IntCal20 and 50% Marine20 (ΔR = -140±35 $^{14}C$ years[21]) with 10% variance. For marine shells, we calibrate the dates based on Marine20 with a ΔR value -140±35 $^{14}C$ years.

## Table S3.3 Calibrated and admixture dates for I41048 under alternative models

| Calibrated Dates (calBP) | | | | | | | |
|---|---|---|---|---|---|---|---|
| Protein | ΔR=–250±50 | ΔR=–140±35 | ΔR=–52±22 | ΔR=0 | ΔR=34±44 | ΔR=75±68 | ΔR=168±43 |
| 0% | -- | -- | -- | 3071-2876 | -- | -- | -- |
| 20% | 3056-2799 | 3001-2789 | 2996-2762 | 2994-2757 | 2993-2753 | 2994-2747 | 2992-2734 |
| 50% | 2965-2750 | **2924-2710** | 2880-2612 | 2862-2541 | 2852-2502 | 2851-2458 | 2776-2377 |
| 100% | 2936-2526 | 2765-2403 | 2684-2339 | 2640-2295 | 2646-2189 | 2614-2102 | 2408-2016 |
| **Admixture Dates (BP)** | | | | | | | |
| Protein | ΔR=–250±50 | ΔR=–140±35 | ΔR=–52±22 | ΔR=0 | ΔR=34±44 | ΔR=75±68 | ΔR=168±43 |
| 0% | -- | -- | -- | 4062-3607 | -- | -- | -- |
| 20% | 4004-3551 | 3982-3522 | 3966-3051 | 3958-3491 | 3953-3486 | 3950-3478 | 3941-3462 |
| 50% | 3941-3481 | **3880-3429** | 3857-3387 | 3837-3334 | 3825-3286 | 3806-3235 | 3751-3182 |
| 100% | 3871-3320 | 3724-3189 | 3635-3094 | 3565-3037 | 3549-2972 | 3552-2883 | 3344-2801 |

**Notes:** Calibrated dates combine IntCal20 and Marine20 assuming 0%, 20%, 50%, and 100% marine dietary protein with ±10% variance. ΔR(s) come from the literature[21–23,58,61,62]. The results presented in the main text are based on assumptions of 50% marine dietary protein with 10% variance, using ΔR = –140±35 $^{14}$C years for the Palau southwest lagoon[21].

## Table S3.4 Two-way *qpAdm* models for ancient people in Palau

| Target | Source1 | Source2 | Ancestry 1 | Ancestry 2 | P Value |
|---|---|---|---|---|---|
| Ucheliungs | Late Unai Guam | New Guinea Highlanders | 0.568±0.009 | 0.432±0.009 | 7.54E-01 |
| Koror | Late Unai Guam | New Guinea Highlanders | 0.593±0.007 | 0.407±0.007 | 1.20E-01 |
| Omedokl | Late Unai Guam | New Guinea Highlanders | 0.600±0.009 | 0.400±0.009 | 5.04E-01 |
| Ngkeklau | Late Unai Guam | New Guinea Highlanders | 0.604±0.008 | 0.396±0.008 | 5.65E-01 |
| Ucheliungs | Latte Guam | New Guinea Highlanders | 0.570±0.008 | 0.430±0.008 | 4.43E-01 |
| Koror | Latte Guam | New Guinea Highlanders | 0.595±0.007 | 0.405±0.007 | 5.84E-02 |
| Omedokl | Latte Guam | New Guinea Highlanders | 0.600±0.008 | 0.400±0.008 | 3.66E-01 |
| Ngkeklau | Latte Guam | New Guinea Highlanders | 0.606±0.008 | 0.394±0.008 | 5.29E-01 |
| Ucheliungs | Lapita | New Guinea Highlanders | 0.581±0.009 | 0.419±0.009 | 1.18E-02 |
| Koror | Lapita | New Guinea Highlanders | 0.607±0.007 | 0.393±0.007 | 2.43E-03 |
| Omedokl | Lapita | New Guinea Highlanders | 0.614±0.009 | 0.386±0.009 | 5.42E-02 |
| Ngkeklau | Lapita | New Guinea Highlanders | 0.619±0.009 | 0.381±0.009 | 2.04E-01 |
| Palau (2900-2500 BP) | Late Unai Guam | New Guinea Highlanders | 0.561±0.011 | 0.439±0.011 | 8.72E-01 |
| Palau (1700-1200 BP) | Late Unai Guam | New Guinea Highlanders | 0.597±0.006 | 0.403±0.006 | 1.65E-01 |
| Palau (700-500 BP) | Late Unai Guam | New Guinea Highlanders | 0.594±0.013 | 0.406±0.013 | 2.29E-01 |
| Palau (present-day) | Late Unai Guam | New Guinea Highlanders | 0.611±0.007 | 0.389±0.007 | 6.56E-01 |
| Palau (2900-2500 BP) | Latte Guam | New Guinea Highlanders | 0.563±0.010 | 0.437±0.010 | 6.53E-01 |
| Palau (1700-1200 BP) | Latte Guam | New Guinea Highlanders | 0.598±0.006 | 0.402±0.006 | 6.49E-02 |
| Palau (700-500 BP) | Latte Guam | New Guinea Highlanders | 0.595±0.012 | 0.405±0.012 | 6.97E-02 |
| Palau (present-day) | Latte Guam | New Guinea Highlanders | 0.618±0.005 | 0.382±0.005 | 1.60E-01 |
| Palau (2900-2500 BP) | Lapita | New Guinea Highlanders | 0.574±0.010 | 0.426±0.010 | 4.51E-02 |
| Palau (1700-1200 BP) | Lapita | New Guinea Highlanders | 0.611±0.006 | 0.389±0.006 | 3.54E-03 |
| Palau (700-500 BP) | Lapita | New Guinea Highlanders | 0.607±0.013 | 0.393±0.013 | 2.17E-02 |
| Palau (present-day) | Lapita | New Guinea Highlanders | 0.625±0.007 | 0.375±0.007 | 9.52E-02 |

**Notes:** For the above models, we used the following groups from diverse places in the world as right outgroups: Australian, Pima, Onge, Japanese, Dai, Ami, Kankanaey, Nasioi, Baining. For the Lapita group, we merged published ancient DNA data from Vanuatu and Tonga aiming to improve data quality.

## Table S3.5 Continuous East Asian-related gene flow into Palau over time

| Target | Source 1 | Source 2 | Proportion1 | Proportion2 | P Value |
|---|---|---|---|---|---|
| Koror | Ucheliungs | Late Unai Guam | 0.945 ± 0.030 | 0.055 ± 0.030 | 1.79E-01 |
| Omedokl | Ucheliungs | Late Unai Guam | 0.910 ± 0.031 | 0.090 ± 0.031 | 2.14E-01 |
| Ngkeklau | Ucheliungs | Late Unai Guam | 0.899 ± 0.029 | 0.101 ± 0.029 | 1.22E-01 |
| Present-day Palau | Ucheliungs | Late Unai Guam | 0.906 ± 0.022 | 0.094 ± 0.022 | 3.58E-01 |
| Koror | Ucheliungs | Latte Guam | 0.943 ± 0.028 | 0.057 ± 0.028 | 1.85E-01 |
| Omedokl | Ucheliungs | Latte Guam | 0.907 ± 0.032 | 0.093 ± 0.032 | 2.25E-01 |
| Ngkeklau | Ucheliungs | Latte Guam | 0.894 ± 0.027 | 0.106 ± 0.027 | 1.52E-01 |
| Present-day Palau | Ucheliungs | Latte Guam | 0.903 ± 0.021 | 0.097 ± 0.021 | 4.28E-01 |
| Koror | Ucheliungs | Lapita | 0.940 ± 0.030 | 0.060 ± 0.030 | 2.01E-01 |
| Omedokl | Ucheliungs | Lapita | 0.903 ± 0.032 | 0.097 ± 0.032 | 2.54E-01 |
| Ngkeklau | Ucheliungs | Lapita | 0.887 ± 0.030 | 0.113 ± 0.030 | 2.19E-01 |
| Present-day Palau | Ucheliungs | Lapita | 0.900 ± 0.023 | 0.100 ± 0.023 | 4.71E-01 |
| Palau (1700-1200 BP) | Palau (2900-2500 BP) | Late Unai Guam | 0.916 ± 0.021 | 0.084 ± 0.021 | 3.79E-01 |
| Palau (700-500 BP) | Palau (2900-2500 BP) | Late Unai Guam | 0.927 ± 0.033 | 0.073 ± 0.033 | 5.68E-01 |
| Palau (present-day) | Palau (2900-2500 BP) | Late Unai Guam | 0.894 ± 0.022 | 0.106 ± 0.022 | 4.28E-01 |
| Palau (1700-1200 BP) | Palau (2900-2500 BP) | Latte Guam | 0.915 ± 0.022 | 0.085 ± 0.022 | 3.91E-01 |
| Palau (700-500 BP) | Palau (2900-2500 BP) | Latte Guam | 0.929 ± 0.035 | 0.071 ± 0.035 | 5.36E-01 |
| Palau (present-day) | Palau (2900-2500 BP) | Latte Guam | 0.892 ± 0.022 | 0.108 ± 0.022 | 4.72E-01 |
| Palau (1700-1200 BP) | Palau (2900-2500 BP) | Lapita | 0.908 ± 0.023 | 0.092 ± 0.023 | 4.88E-01 |
| Palau (700-500 BP) | Palau (2900-2500 BP) | Lapita | 0.924 ± 0.036 | 0.076 ± 0.036 | 5.45E-01 |
| Palau (present-day) | Palau (2900-2500 BP) | Lapita | 0.888 ± 0.024 | 0.112 ± 0.024 | 4.96E-01 |

**Notes:** For the above models, we used the following groups from diverse places in the world as the outgroups: Australian, Pima, Onge, Japanese, Dai, Ami, Kankanaey, Nasioi, Baining, and New Guinea Highlanders. For the Lapita group, we merged published ancient DNA data from Vanuatu and Tonga to increase resolution.

## Table S3.6 Estimation of sex-biased admixture

| Target | N | Source1 | Source2 | Autosomal SNP | | X chromosomal SNP | | Diff. | |
|---|---|---|---|---|---|---|---|---|---|
| | | | | East Asian | Papuan | East Asian | Papuan | Mean | Z |
| Ucheliungs | 3 | Kankanaey | New Guinea Highlander | 0.565±0.009 | 0.435±0.009 | 0.725±0.064 | 0.275±0.064 | 0.160±0.065 | 2.476 |
| Koror Quarry | 11 | Kankanaey | New Guinea Highlander | 0.589±0.007 | 0.411±0.007 | 0.745±0.043 | 0.255±0.043 | 0.156±0.044 | 3.581 |
| Omedokl | 3 | Kankanaey | New Guinea Highlander | 0.599±0.009 | 0.401±0.009 | 0.723±0.068 | 0.277±0.068 | 0.124±0.069 | 1.808 |
| Ngkeklau | 3 | Kankanaey | New Guinea Highlander | 0.603±0.009 | 0.397±0.009 | 0.817±0.051 | 0.183±0.051 | 0.214±0.052 | 4.132 |
| Palau (2900-2500 BP) | 2 | Kankanaey | New Guinea Highlander | 0.556±0.011 | 0.444±0.011 | 0.751±0.070 | 0.249±0.070 | 0.195±0.071 | 2.752 |
| Palau (1700-1200 BP) | 14 | Kankanaey | New Guinea Highlander | 0.595±0.006 | 0.404±0.006 | 0.763±0.044 | 0.237±0.044 | 0.168±0.044 | 3.783 |
| Palau (1600-1200 BP) | 6 | Kankanaey | New Guinea Highlander | 0.599±0.008 | 0.401±0.008 | 0.783±0.049 | 0.217±0.049 | 0.184±0.050 | 3.706 |

**Notes:** Ancestry was estimated by *qpAdm* on autosomal and X-chromosomal SNPs, separately. N, sample size for each target group.

## Table S3.7 Dates of admixture

| | | | Admixture Dates 95% CI (BP) | | | |
|---|---|---|---|---|---|---|
| Target (Admixed) | Admixture Time (Generations) | Z | 0% Marine Protein | 20% Marine Protein | 50% Marine Protein | 100% Marine Protein |
| Ucheliungs (I41048 only) | 30.126±3.513 | 8.577 | 4062-3607 | 3985-3544 | 3880-3429 | 3727-3188 |
| Ucheliungs (I41049 only) | 27.516±6.210 | 4.431 | 3951-3244 | 3917-3202 | 3819-3056 | 3634-2839 |
| Ucheliungs (I41048 and I41049) | 29.161±3.821 | 7.632 | 4023-3465 | 3959-3433 | 3864-3213 | 3699-2997 |
| Koror Quarry (N=11) | 57.623±5.753 | 10.016 | 3702-2995 | 3625-2924 | 3543-2778 | 3365-2572 |
| Omedokl (N=3) | 54.485±9.075 | 6.004 | 3535-2014 | 3507-1967 | 3398-1837 | 3247-1611 |
| Ngkeklau (N=3) | 50.967±4.929 | 10.341 | 3339-2680 | 3323-2656 | 3208-2523 | 3045-2294 |
| Palau (1600-1200 BP) (N=6) | 51.366±4.281 | 11.997 | 3324-2721 | 3306-2696 | 3194-2523 | 3036-2330 |
| Palau (1700-1200 BP) (N=17) | 54.167±3.728 | 14.529 | 3478-2836 | 3405-2804 | 3346-2630 | 3172-2434 |
| Omedokl (I41045) (700-500 BP) (N=1) | 79.349±13.921 | 5.700 | 3833-2256 | 3763-2194 | 3674-2083 | 3513-1924 |
| Palau (present-day) | 85.062±6.408 | 13.274 | 2791-2040 | 2791-2040 | 2791-2040 | 2791-2040 |

**Notes:** We merged 56 individuals of Kankanaey, Ami, Atayal, Dai, and Han to estimate East Asian allele frequencies, and we used 25 individuals of New Guinea Highlanders, Middle Sepik Papuans, and Nasioi speakers to estimate Papuan allele frequencies. We assumed the generation interval of modern human to be 28.4 years in average, with a 95% CI of 27-30 years[19]. For the propagation of uncertainty, see Table S4.3. The "Palau (1600-1200 BP)" group includes one Ucheliungs, two Omedokl, and three Ngkeklau individuals; the "Palau (1700-1200 BP)" group includes Palau group one and eleven Koror Quarry individuals dating to 1700-1200 BP. Calibrated dates combine IntCal20 and Marine20 assuming 0%, 20%, 50%, and 100% marine dietary protein with ±10% variance. ΔR = -140 ± 35 $^{14}C$ years[21] is applied.

## Table S3.8 Genetic link between Palau and Northeastern Indonesia

| Target Population (Admixed) | Source 1 | Source 2 | Proportion1 ± Std.Err | Proportion2 ± Std.Err | P Value |
|---|---|---|---|---|---|
| Ucheliungs | Morotai 2100BP | New Guinea Highlander | 0.834 ± 0.016 | 0.166 ± 0.016 | 8.44E-01 |
| Koror Quarry | Morotai 2100BP | New Guinea Highlander | 0.873 ± 0.017 | 0.127 ± 0.017 | 2.42E-01 |
| Omedokl | Morotai 2100BP | New Guinea Highlander | 0.891 ± 0.016 | 0.109 ± 0.016 | 2.57E-01 |
| Ngkeklau | Morotai 2100BP | New Guinea Highlander | 0.890 ± 0.017 | 0.110 ± 0.017 | 4.45E-01 |
| Palau (2900-2700 BP) | Morotai 2100BP | New Guinea Highlander | 0.823 ± 0.016 | 0.177 ± 0.016 | 9.54E-01 |
| Palau (1700-1200 BP) | Morotai 2100BP | New Guinea Highlander | 0.877 ± 0.011 | 0.123 ± 0.011 | 2.82E-01 |
| Palau (700-500 BP) | Morotai 2100BP | New Guinea Highlander | 0.873 ± 0.019 | 0.127 ± 0.019 | 4.54E-01 |
| Morotai 2100BP | Palau (2900-2700 BP) | Late Unai Guam | 0.727 ± 0.021 | 0.273 ± 0.021 | 8.22E-01 |
| Morotai 2100BP | Palau (2900-2700 BP) | Latte Guam | 0.725 ± 0.022 | 0.275 ± 0.022 | 8.15E-01 |
| Morotai 2100BP | Palau (2900-2700 BP) | Lapita | 0.716 ± 0.022 | 0.284 ± 0.022 | 3.88E-01 |
| Morotai 2100BP | Ancient Palau | Late Unai Guam | 0.803 ± 0.020 | 0.197 ± 0.020 | 3.96E-01 |
| Morotai 2100BP | Ancient Palau | Latte Guam | 0.803 ± 0.020 | 0.197 ± 0.020 | 2.68E-01 |
| Morotai 2100BP | Ancient Palau | Lapita | 0.800 ± 0.021 | 0.200 ± 0.021 | 3.61E-02 |

**Notes:** We used the following groups from diverse places in the world as the outgroups: Australian, Pima, Onge, Japanese, Dai, Ami, Kankanaey, Nasioi, Baining, and New Guinea Highlanders. For the Lapita group, we merged published ancient DNA data from Vanuatu and Tonga to increase resolution. When we used ancient Morotai from Indonesia as a source, models succeed even with Lapita or Guam Late Unai in the outgroups. These estimates are based on autosomal SNPs.

## Supplemental Reference

1. Lipson, M., Loh, P.-R., Patterson, N., Moorjani, P., Ko, Y.-C., Stoneking, M., Berger, B., and Reich, D. (2014). Reconstructing Austronesian population history in island Southeast Asia. Nat. Commun. *5*, 4689. 10.1038/ncomms5689.
2. Skoglund, P., Posth, C., Sirak, K., Spriggs, M., Valentin, F., Bedford, S., Clark, G.R., Reepmeyer, C., Petchey, F., Fernandes, D., et al. (2016). Genomic insights into the peopling of the Southwest Pacific. Nature. *538*, 510-513. 10.1038/nature19844.
3. Lipson, M., Skoglund, P., Spriggs, M., Valentin, F., Bedford, S., Shing, R., Buckley, H., Phillip, I., Ward, G.K., Mallick, S., et al. (2018). Population turnover in Remote Oceania shortly after initial settlement. Curr. Biol. *28*, 1157–1165. 10.1016/j.cub.2018.02.051.
4. Posth, C., Nägele, K., Colleran, H., Valentin, F., Bedford, S., Kami, K.W., Shing, R., Buckley, H., Kinaston, R., Walworth, M., et al. (2018). Language continuity despite population replacement in Remote Oceania. Nat. Ecol. Evol. *2*, 731-740. 10.1038/s41559-018-0498-2.
5. Lipson, M., Spriggs, M., Valentin, F., Bedford, S., Shing, R., Zinger, W., Buckley, H., Petchey, F., Matanik, R., Cheronet, O., et al. (2020). Three Phases of Ancient Migration Shaped the Ancestry of Human Populations in Vanuatu. Curr. Biol. *30*, 4846-4856. 10.1016/j.cub.2020.09.035.
6. Liu, Y.-C., Hunter-Anderson, R., Cheronet, O., Eakin, J., Camacho, F., Pietrusewsky, M., Rohland, N., Ioannidis, A., Athens, J.S., and Douglas, M.T. (2022). Ancient DNA Reveals Five Streams of Migration into Micronesia and Matrilocality in Early Pacific Seafarers. Science *377*, 72–79. 10.1126/science.abm6536.
7. Choin, J., Mendoza-Revilla, J., Arauna, L.R., Cuadros-Espinoza, S., Cassar, O., Larena, M., Ko, A.M.-S., Harmant, C., Laurent, R., Verdu, P., et al. (2021). Genomic insights into population history and biological adaptation in Oceania. Nature *592*, 583–589. 10.1038/s41586-021-03236-5.
8. Pugach, I., Hübner, A., Hung, H., Meyer, M., Carson, M.T., and Stoneking, M. (2021). Ancient DNA from Guam and the peopling of the Pacific. Proc. Natl. Acad. Sci. 118, e2022112118. 10.1073/pnas.2022112118.
9. Matisoo-Smith, E., and Gosling, A.L. (2025). Understanding the human settlement of the Pacific—Are we there yet? Journal of Archaeological Science, *180*, 106307. 10.1016/j.jas.2025.106307.
10. Yang, M.A., Fan, X., Sun, B., Chen, C., Lang, J., Ko, Y.-C., Tsang, C., Chiu, H., Wang, T., Bao, Q., et al. (2020). Ancient DNA indicates human population shifts and admixture in northern and southern China. Science *369*, 282-288. 10.1126/science.aba0909.

11. Wang, C.-C., Yeh, H.-Y., Popov, A.N., Zhang, H.-Q., Matsumura, H., Sirak, K., Cheronet, O., Kovalev, A., Rohland, N., Kim, A.M., et al. (2021). Genomic insights into the formation of human populations in East Asia. Nature *591*, 413–419. 10.1038/s41586-021-03336-2.
12. O'Connell, J.F., and Allen, J. (2015). The process, biotic impact, and global implications of the human colonization of Sahul about 47,000 years ago. J. Archaeol. Sci. *56*, 73–84. 10.1016/j.jas.2015.02.020.
13. Wickler, S., and Spriggs, M. (1988). Pleistocene human occupation of the Solomon Islands. Antiquity *62*, 703–706. 10.1017/S0003598X00075104.
14. Moore, D.R., and Hunter-Anderson, R.L. (1999). Pots and pans in the intermediate Pre-Latte (2500-1600 bp), Mariana islands, Micronesia. In The Pacific from 5000 to 2000 BP: Colonisation and Transformations, J.-C. Galipaud and I. Lilley, ed. (IRD Editions), pp.487–503.
15. Blust, R. (2013). The Austronesian languages (revised edition) (ANU Press).
16. Ross, M., Pawley, A., and Osmond, M. (2023). The Lexicon of Proto Oceanic. The Culture and Environment of Ancestral Oceanic Society. Volume 6: People: Society. (Canberra: The Australian National University).
17. Spriggs, M., Geraghty, P., and Liu, Y.-C. (2025). Austronesian archaeolinguistics. In the Oxford Handbook of Archaeology and Language, M. Robbeets and M. Hudson, ed. (Oxford: Oxford University Press), pp. 466–492.
18. Stone, J.H. (2020). The Bioarchaeology of Initial Human Settlement in Palau, Western Micronesia (University of Oregon, Ph.D. thesis).
19. Iasi, L.N.M., Chintalapati, M., Skov, L., Mesa, A.B., Hajdinjak, M., Peter, B.M., and Moorjani, P. (2024). Neandertal ancestry through time: Insights from genomes of ancient and present-day humans. Science *386*, eadq3010. 10.1126/science.adq3010.
20. Clark, G.R. (2005). A 3000-year culture sequence from Palau, western Micronesia. Asian Perspect., *44*, 349–380. 10.1353/asi.2005.0020
21. Clark, G., Anderson, A., and Wright, D. (2006). Human colonization of the Palau Islands, western Micronesia. J. Isl. & Coast. Archaeol. *1*, 215–232. 10.1080/15564890600831705.
22. Masse, W.B., Liston, J., Carucci, J., and Athens, J.S. (2006). Evaluating the effects of climate change on environment, resource depletion, and culture in the Palau Islands between AD 1200 and 1600. Quat. Int. *151*, 106–132. 10.1016/j.quaint.2006.01.017.
23. Petchey, F., and Clark, G. (2010). A ΔR for the Palau Islands: An evaluation of extant and new ΔR values and their application to archaeological deposits at Ulong. J. Isl. Archaeol. *5*, 236–252. 10.1080/15564890903155935.

24. Stone, J.H., Fitzpatrick, S.M., and Krigbaum, J. (2019). Isotopic Analysis of Prehistoric Human Diet at Chelechol ra Orrak, Palau. Bioarchaeology International *3*, 142–156. 10.5744/bi.2019.1009.
25. Oliveira, S., Nägele, K., Carlhoff, S., Pugach, I., Koesbardiati, T., Hübner, A., Meyer, M., Oktaviana, A.A., Takenaka, M., Katagiri, C., et al. (2022). Ancient genomes from the last three millennia support multiple human dispersals into Wallacea. Nat. Ecol. & Evol., *6*, 1024–1034. 10.1038/s41559-022-01775-2.
26. Bellwood, P. (1998). The archaeology of Papuan and Austronesian prehistory in the northern Moluccas, eastern Indonesia. In Archaeol. Lang. II Correl. Archaeol. Linguist. hypotheses, R. Blench, M. Spriggs, ed. (London: Routledge), pp. 128–140.
27. Bellwood, P. (2019). The Spice Islands in Prehistory: Archaeology in the Northern Moluccas, Indonesia (ANU Press).
28. Lin, S., Dong, B., and Yang, S. (2024). Enhanced impacts of ENSO on the Southeast Asian summer monsoon under global warming and associated mechanisms. Geophys. Res. Lett. *51*, e2023GL106437. 10.1029/2023GL106437.
29. Lu, F., and Liu, Z. (2018). Assessing extratropical influence on observed El Niño--Southern Oscillation events using regional coupled data assimilation. J. Clim. *31*, 8961–8969. 10.1175/JCLI-D-17-0849.1.
30. Callaghan, R., and Fitzpatrick, S.M. (2008). Examining prehistoric migration patterns in the Palauan archipelago: a computer simulated analysis of drift voyaging. Asian Perspect., *47*, 28–44. 10.1353/asi.2008.0007.
31. Montenegro, Á., Callaghan, R.T., and Fitzpatrick, S.M. (2016). Using seafaring simulations and shortest-hop trajectories to model the prehistoric colonization of Remote Oceania. Proc. Natl. Acad. Sci. *113*, 12685–12690. 10.1073/pnas.1612426113.
32. Liston, J., Hoerman, R., Sasao, M.O., and Kloulubak, S. (2021). A review of the rock art of Palau, Micronesia in local and regional contexts. Archaeol. Ocean. *56*, 322–343. 10.1002/arco.5254.
33. Liston, J. (2009). Cultural chronology of earthworks in Palau, western Micronesia. Archaeol. Ocean. *44*, 56–73. 10.1002/J.1834-4453.2009.TB00047.X.
34. Sirak, K.A., Fernandes, D.M., Cheronet, O., Novak, M., Gamarra, B., Balassa, T., Bernert, Z., Cséki, A., Dani, J., Gallina, J.Z., et al. (2017). A minimally-invasive method for sampling human petrous bones from the cranial base for ancient DNA analysis. Biotechniques *62*, 283–289. 10.2144/000114558.
35. Pinhasi, R., Fernandes, D.M., Sirak, K., and Cheronet, O. (2019). Isolating the human cochlea to generate bone powder for ancient DNA analysis. Nat. Protoc. *14*, 1194–1205. 10.1038/s41596-019-0137-7.

36. Dabney, J., Knapp, M., Glocke, I., Gansauge, M.-T., Weihmann, A., Nickel, B., Valdiosera, C., García, N., Pääbo, S., Arsuaga, J.-L., et al. (2013). Complete mitochondrial genome sequence of a Middle Pleistocene cave bear reconstructed from ultrashort DNA fragments. Proc. Natl. Acad. Sci. U.S.A. *110*, 15758-15763. 10.1073/pnas.1314445110.
37. Rohland, N., Harney, E., Mallick, S., Nordenfelt, S., and Reich, D. (2015). Partial uracil-DNA-glycosylase treatment for screening of ancient DNA. Philos. Trans. R. Soc. B Biol. Sci. *370*, 20130624. 10.1098/rstb.2013.0624.
38. Rohland, N., Mallick, S., Mah, M., Maier, R.M., Patterson, N.J., and Reich, D. (2022). Three assays for in-solution enrichment of ancient human DNA at more than a million SNPs. Genome Res. *32*, 2068–2078. 10.1101/gr.276728.122.
39. Fu, Q., Mittnik, A., Johnson, P.L.F., Bos, K., Lari, M., Bollongino, R., Sun, C., Giemsch, L., Schmitz, R., Burger, J., et al. (2013). A revised timescale for human evolution based on ancient mitochondrial genomes. Curr. Biol. *23*, 553–559. 10.1016/j.cub.2013.02.044.
40. Furtwängler, A., Reiter, E., Neumann, G.U., Siebke, I., Steuri, N., Hafner, A., Lösch, S., Anthes, N., Schuenemann, V.J., and Krause, J. (2018). Ratio of mitochondrial to nuclear DNA affects contamination estimates in ancient DNA analysis. Sci. Rep. *8*, 1–8. 10.1038/s41598-018-32083-0.
41. Korneliussen, T.S., Albrechtsen, A., and Nielsen, R. (2014). ANGSD: analysis of next generation sequencing data. BMC Bioinformatics *15*, 356. 10.1186/s12859-014-0356-4.
42. Weissensteiner, H., Pacher, D., Kloss-Brandstätter, A., Forer, L., Specht, G., Bandelt, H.-J., Kronenberg, F., Salas, A., and Schönherr, S. (2016). HaploGrep 2: mitochondrial haplogroup classification in the era of high-throughput sequencing. Nucleic Acids Res. *44*, W58-W63. 10.1093/nar/gkw233.
43. Alaçamlı, E., Naidoo, T., Güler, M.N., Sağlıcan, E., Aktürk, S., Mapelli, I., Vural, K.B., Somel, M., Malmström, H. and Günther, T. (2024). READv2: advanced and user-friendly detection of biological relatedness in archaeogenomics. Genome Biol *25*, 216. 10.1186/s13059-024-03350-3.
44. Heaton, T.J., Köhler, P., Butzin, M., Bard, E., Reimer, R.W., Austin, W.E.N., Ramsey, C.B., Grootes, P.M., Hughen, K.A., Kromer, B., et al. (2020). Marine20—the marine radiocarbon age calibration curve (0--55,000 cal BP). Radiocarbon *62*, 779–820. 10.1017/RDC.2020.68.
45. Hughen, K.A., Baillie, M.G.L., Bard, E., Beck, J.W., Bertrand, C.J.H., Blackwell, P.G., Buck, C.E., Burr, G.S., Cutler, K.B., Damon, P.E., et al. (2004). Marine04 marine radiocarbon age calibration, 0-26 cal kyr BP. Radiocarbon *46*, 1059–1086. 10.1017/S0033822200033002.

46. Reimer, P.J., Baillie, M.G.L., Bard, E., Bayliss, A., Beck, J.W., Blackwell, P.G., Ramsey, C.B., Buck, C.E., Burr, G.S., Edwards, R.L., et al. (2009). IntCal09 and Marine09 radiocarbon age calibration curves, 0-50,000 years cal BP. Radiocarbon *51*, 1111–1150. 10.1017/S0033822200034202.

47. Reimer, P.J., Baillie, M.G.L., Bard, E., Bayliss, A., Beck, J.W., Blackwell, P.G., Ramsey, C.B., Buck, C.E., Burr, G.S., Edwards, R.L., et al. (2013). IntCal13 and Marine13 radiocarbon age calibration curves 0-50,000 years cal BP. Radiocarbon *55*, 1869–1887. 10.2458/azu_js_rc.55.16947.

48. Stuiver, M., Pearson, G.W., and Braziunas, T. (1986). Radiocarbon age calibration of marine samples back to 9000 cal yr BP. Radiocarbon *28*, 980–1021. 10.1017/S0033822200060264.

49. Alves, E., Macario, K., Souza, R., Aguilera, O., Goulart, A.C., Scheel-Ybert, R., Bachelet, C., Carvalho, C., Oliveira, F., and Douka, K. (2015). Marine reservoir corrections on the southeastern coast of Brazil: paired samples from the Saquarema shellmound. Radiocarbon *57*, 517–525. 10.2458/azu_rc.57.18404.

50. Deviese, T., Stafford Jr, T.W., Waters, M.R., Wathen, C., Comeskey, D., Becerra-Valdivia, L., and Higham, T. (2018). Increasing accuracy for the radiocarbon dating of sites occupied by the first Americans. Quat. Sci. Rev. *198*, 171–180. 10.1016/j.quascirev.2018.08.023.

51. Patterson, N., Price, A.L., and Reich, D. (2006). Population structure and eigenanalysis. PLoS Genet. *2*, 2074–2093. 10.1371/journal.pgen.0020190.

52. Patterson, N., Moorjani, P., Luo, Y., Mallick, S., Rohland, N., Zhan, Y., Genschoreck, T., Webster, T., and Reich, D. (2012). Ancient admixture in human history. Genetics *192*, 1065–1093. 10.1534/genetics.112.145037.

53. Haak, W., Lazaridis, I., Patterson, N., Rohland, N., Mallick, S., Llamas, B., Brandt, G., Nordenfelt, S., Harney, E., Stewardson, K., et al. (2015). Massive migration from the steppe was a source for Indo-European languages in Europe. Nature *522*, 207–211. 10.1038/nature14317.

54. Narasimhan, V.M., Patterson, N., Moorjani, P., Rohland, N., Bernardos, R., Mallick, S., Lazaridis, I., Nakatsuka, N., Olalde, I., Lipson, M., et al. (2019). The formation of human populations in South and Central Asia. Science *365*, eaat7487. 10.1126/science.aat7487.

55. Chintalapati, M., Patterson, N., and Moorjani, P. (2022). The spatiotemporal patterns of major human admixture events during the European Holocene. Elife *11*, e77625. 10.7554/eLife.77625.

56. Akbari, A., Barton, A.R., Gazal, S., Li, Z., Kariminejad, M., Perry, A., Zeng, Y., Mittnik, A., Patterson, N., Mah, M., et al. (2024). Pervasive findings of directional selection realize the promise of ancient DNA to elucidate human adaptation. bioRxiv. 10.1101/2024.09.14.613021.

57. Fitzpatrick, S.M., and Jew, N.P. (2018). Radiocarbon dating and Bayesian modelling of one of Remote Oceania's oldest cemeteries at Chelechol ra Orrak, Palau. Antiquity *92*, 149–164. 10.15184/aqy.2017.229.
58. Stone, J.H., Fitzpatrick, S.M., and Napolitano, M.F. (2017). Disproving claims for small-bodied humans in the Palauan archipelago. Antiquity *91*, 1546–1560. 10.15184/aqy.2017.184.
59. Liston, J. (2005). An assessment of radiocarbon dates from Palau, western Micronesia. Radiocarbon *47*, 295–354. 10.1017/S0033822200019780.
60. Fitzpatrick, S.M. (2003). Early human burials in the western Pacific: evidence for c. 3000 year old occupation on Palau. Antiquity *77*, 719–731. 10.1017/S0003598X00061664.
61. Fitzpatrick, S.M. (2002). A radiocarbon chronology of Yapese stone money quarries in Palau. Micronesica *34*, 227–242.
62. Yoneda, M., Uno, H., Shibata, Y., Suzuki, R., Kumamoto, Y., Yoshida, K., Sasaki, T., Suzuki, A., and Kawahata, H. (2007). Radiocarbon marine reservoir ages in the western Pacific estimated by pre-bomb molluscan shells. Nucl. Instruments Methods Phys. Res. Sect. B Beam Interact. with Mater. Atoms *259*, 432–437. 10.1016/j.nimb.2007.01.184.
63. Clark, G., and Reepmeyer, C. (2012). Last millennium climate change in the occupation and abandonment of Palau's Rock Islands. Archaeol. Ocean. *47*, 29–38. 10.1002/j.1834-4453.2012.tb00112.x.
64. Liston, J. (2013). Sociopolitical development and a monumental earthwork landscape on Babeldaob Island, Palau (Australian National University).
65. Dickinson, W.R., and Athens, J.S. (2007). Holocene paleoshoreline and paleoenvironmental history of Palau: Implications for human settlement. J. Isl. Coast. Archaeol. *2*, 175–196. 10.1080/15564890701623639.
66. Fitzpatrick, S.M., Giovas, C.M., and Kataoka, O. (2011). Temporal trends in prehistoric fishing in Palau, Micronesia over the last 1500 years. Archaeol. Ocean. *46*, 6–16. 10.1002/j.1834-4453.2011.tb00094.x.
67. Stone, J.H., Fitzpatrick, S.M., and Krigbaum, J. (2019). Stable isotope analysis of human diet at Chelechol ra Orrak, Palau. Bioarchaeology Int. *3*, 142-156. 10.5744/bi.2019.1009.
68. Mueller-Dombois, D. and Fosberg, F.R. (1998). Vegetation of the Tropical Pacific Islands. (Springer-Verlag New York, Inc.).
69. Athens, J.S. and Ward, J.V. (2005). Palau Compact Road Archaeological Investigations, Babeldaob Island, Republic of Palau; Phase I: Intensive Archaeological Survey; Volume IV: Holocene

Paleoenvironment and Landscape Change. Prepared for the U.S. Army Corps of Engineers, Pacific Ocean Division, Hawai'i. International Archaeological Research Institute, Inc., Honolulu.

70. Liston, J. and Iida, A. (2020). Legacies on Babeldaob's landscape. In Ethnobotany of Palau: Plants, People, and Island Culture, Volume I. M.J. Balickand A. Kitalong, ed. (Belau National Museum and the New York Botanical Garden) pp.55-94.
71. Athens, J.S. and Stevenson, J. (2012). Pohnpei coring records: The natural distribution of *Cyrtosperma chamissonis*. The Journal of Pacific Archaeology *3*, 35-48. 10.70460/jpa.v3i1.75.
72. De Langhe, E., L. Vrydaghs, P. de Maret, X. Perrier, and T. Denham. (2009). Why bananas matter: An introduction to the history of banana domestication. Ethnobotany Research and Applications *7*, 165-177. 10.17348/era.7.0.165-177.
73. Zerega, N., D. Ragone, and T. Motley. (2004). Complex origins of breadfruit (*Artocarpus altilis*, Moraceae): Implications for human migrations in Oceania. American Journal of Botany *91*, 760-766. 10.3732/ajb.91.5.760.
74. G. R. Clark, W. D. (2007). Reading Pacific pots. In Vastly Ingenious: The Archaeology of Pacific Material Culture: in Honour of Janet M. Davidson. A. Anderson, K. Green, and F. Leach, ed. (University of Otago Press, New Zealand) pp. 173-189.
75. Reepmeyer, C., Clark, G., Liston, J., and Ussher, E. (2016). On the importance of freshwater access in successful island colonisation. In Spatial Dynamics in Oceania Discovery, Appropriation and the Emergence of Traditional Societies, F. Valentin and G. Molle, ed. (Société préhistorique française) pp. 49–62.
76. Liston, J., Rieth, T.M., and HD, T. (2011). Traditional pottery analysis. In Archaeological Monitoring and Emergency Data Recovery for the Compact Road, Babeldaob Island, Republic of Palau. Historic Preservation Investigations, Phase III. Volume 12: Lab Analyses, Discussion, Syntheses (International Archaeological Research Institute). pp. 262-332.
77. Liston, J., and Tuggle, H.D. (2006). Prehistoric warfare in Palau. In The Archaeology of Warfare: Prehistories of Raiding and Conquest, E. Arkush and M. W. Allen, ed. (University Press of Florida, Gainesville) pp 148-183.
78. Farley, G., Schneider, L., Clark, G., and Haberle, S.G. (2018). A Late Holocene palaeoenvironmental reconstruction of Ulong Island, Palau, from starch grain, charcoal, and geochemistry analyses. J. Archaeol. Sci. Reports *22*, 248–256. 10.1016/j.jasrep.2018.09.024.
79. Intoh, M., and Dickinson, W.R. (2002). Prehistoric pottery movements in western Micronesia: technological and petrological study of potsherds from Fais Island. In Fifty Years in the Field: Essays in

Honour and Celebration of Richard Shutler Jr's Archaeological Career, S. Bedford, C. Sand, and D. Burley, ed. (Auckland: New Zealand Archaeological Association Monograph 25) pp. 123-134.

80. Smittenberg, R.H., Saenger, C., Dawson, M.N., and Sachs, J.P. (2011). Compound-specific D/H ratios of the marine lakes of Palau as proxies for West Pacific Warm Pool hydrologic variability. Quat. Sci. Rev. *30*, 921–933. 10.1016/j.quascirev.2011.01.012.
81. Sachs, J.P., Blois, J.L., McGee, T., Wolhowe, M., Haberle, S., Clark, G., and Atahan, P. (2018). Southward shift of the Pacific ITCZ during the Holocene. Paleoceanography and Paleoclimatology *33*, 1383–1395. 10.1029/2018PA003469.
82. Fitzpatrick, S.M., and Nelson, G.C. (2008). From limestone caves to concrete graves: 3000 years of mortuary practice in the Palauan archipelago, Micronesia. Int. J. Osteoarchaeol. *18*, 439–457. 10.1002/oa.951.
83. Liston, J. (2014). Ritual use of Palau's monumental earthworks and leadership strategies. In Monuments and People in the Pacific, H. Martinsson-Wallin and T. Thomas, ed. (Studies in Global Archaeology No. 20, Uppsala University, Visby, Sweden) pp. 101-128.
84. Rieth, T.M., and Liston, J. (2001). Archaeological Data Recovery at Ngermereues Ridge, Ngesaol, Koror, Republic of Palau. (International Archaeological Research Institute).
85. Nelson, G.C., and Fitzpatrick, S.M. (2006). Preliminary investigations of the Chelechol ra Orrak cemetery, Republic of Palau: I, skeletal biology and paleopathology. Anthropol. Sci. *114*, 1–12. 10.1537/ase.040710.
86. Osborne, D. (1966). The archaeology of the Palau Islands: An intensive survey. (Bishop Museum Bulletin 230, Bishop Museum Press, Honolulu).
87. Berger, L.R., Churchill, S.E., De Klerk, B., and Quinn, R.L. (2008). Small-bodied humans from Palau, Micronesia. PLoS One *3*, e1780. 10.1371/journal.pone.0001780.
88. De Klerk, B. (2012). Size variation and body proportions in an isolated Holocene-aged population of Hominids from Palau, Micronesia and its impact on our understanding of variation in extinct hominids (University of the Witwatersrand, Johannesburg, South Africa).
89. Collins, S. (1994). Memorandum: Human Remains from Belau. Bernice P. Bishop Museum, Honolulu.
