# Supplementary material for: Papuan Admixture Predated the Settlement of Palau
Source: Cell. Author manuscript; Available in PMC 2026 Mar 28. (PMC13025617; doi:10.1016/j.cell.2026.02.011)
Supplement: FigureS1_Chronology — Figure S1. Chronology for the sites reported in this study, related to Table 1. Eleven newly reported radiocarbon dates in this study (dark grey) are plotted with 18 previously published dates (14 of human bones and four of marine shells) from four sites. The sources of published data are summarized in Table S3.2 and Supplemental Information. The date ranges shown in this figure represent the 95.4% confidence intervals of the calibrated dates. We recalibrated the published dates. For human bones, we calibrate these dates based on a mixed calibration curve combining 50% IntCal20 and 50% Marine20 (ΔR=−140±35 14C years) with 10% variance. For marine shells, we calibrate the dates based on Marine20 with a ΔR=−140±35 14C years. Each date is labeled using the AMS lab code as the primary identifier, followed by the specimen code in brackets. If either piece of information is unavailable for a given published date, the available identifier is used, with specimen codes consistently enclosed in brackets. [file NIHMS2150805-supplement-FigureS1_Chronology.pdf]

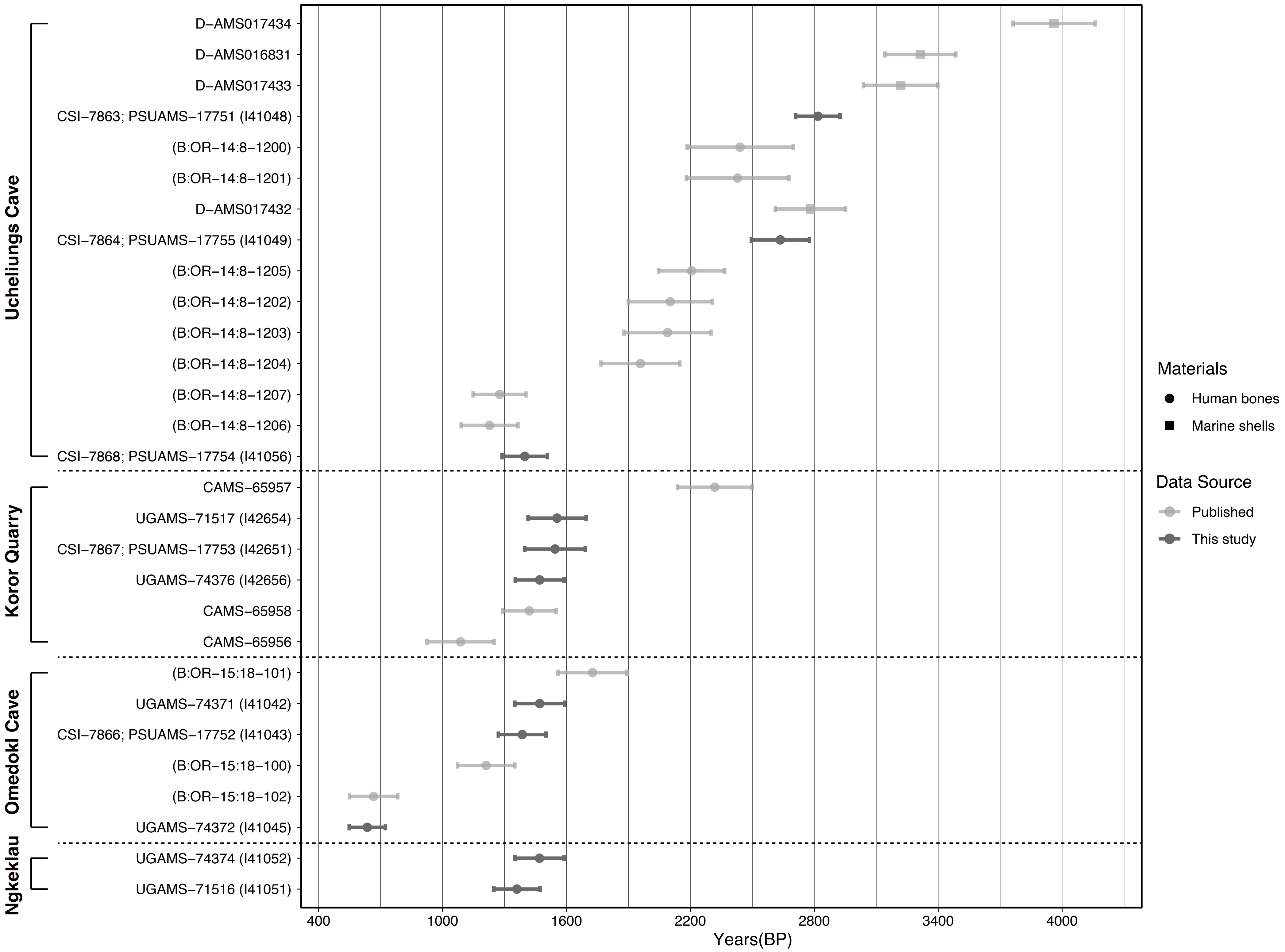
Ucheliungs Cave
D−AMS017434
D−AMS016831
D−AMS017433
CSI−7863; PSUAMS−17751 (I41048)
(B:OR−14:8−1200)
(B:OR−14:8−1201)
D−AMS017432
CSI−7864; PSUAMS−17755 (I41049)
(B:OR−14:8−1205)
(B:OR−14:8−1202)
(B:OR−14:8−1203)
(B:OR−14:8−1204)
(B:OR−14:8−1207)
(B:OR−14:8−1206)
CSI−7868; PSUAMS−17754 (I41056)
Koror Quarry
CAMS−65957
UGAMS−71517 (I42654)
CSI−7867; PSUAMS−17753 (I42651)
UGAMS−74376 (I42656)
CAMS−65958
CAMS−65956
Omedokl Cave
(B:OR−15:18−101)
UGAMS−74371 (I41042)
CSI−7866; PSUAMS−17752 (I41043)
(B:OR−15:18−100)
(B:OR−15:18−102)
UGAMS−74372 (I41045)
Ngkeklau
UGAMS−74374 (I41052)
UGAMS−71516 (I41051)
400
1000
1600
2200
2800
3400
4000
Years(BP)
Materials
Human bones
Marine shells
Data Source
Published
This study
