# Supplementary figures and images for: Papuan Admixture Predated the Settlement of Palau
Source: Cell. Author manuscript; Available in PMC 2026 Mar 28. (PMC13025617; doi:10.1016/j.cell.2026.02.011)

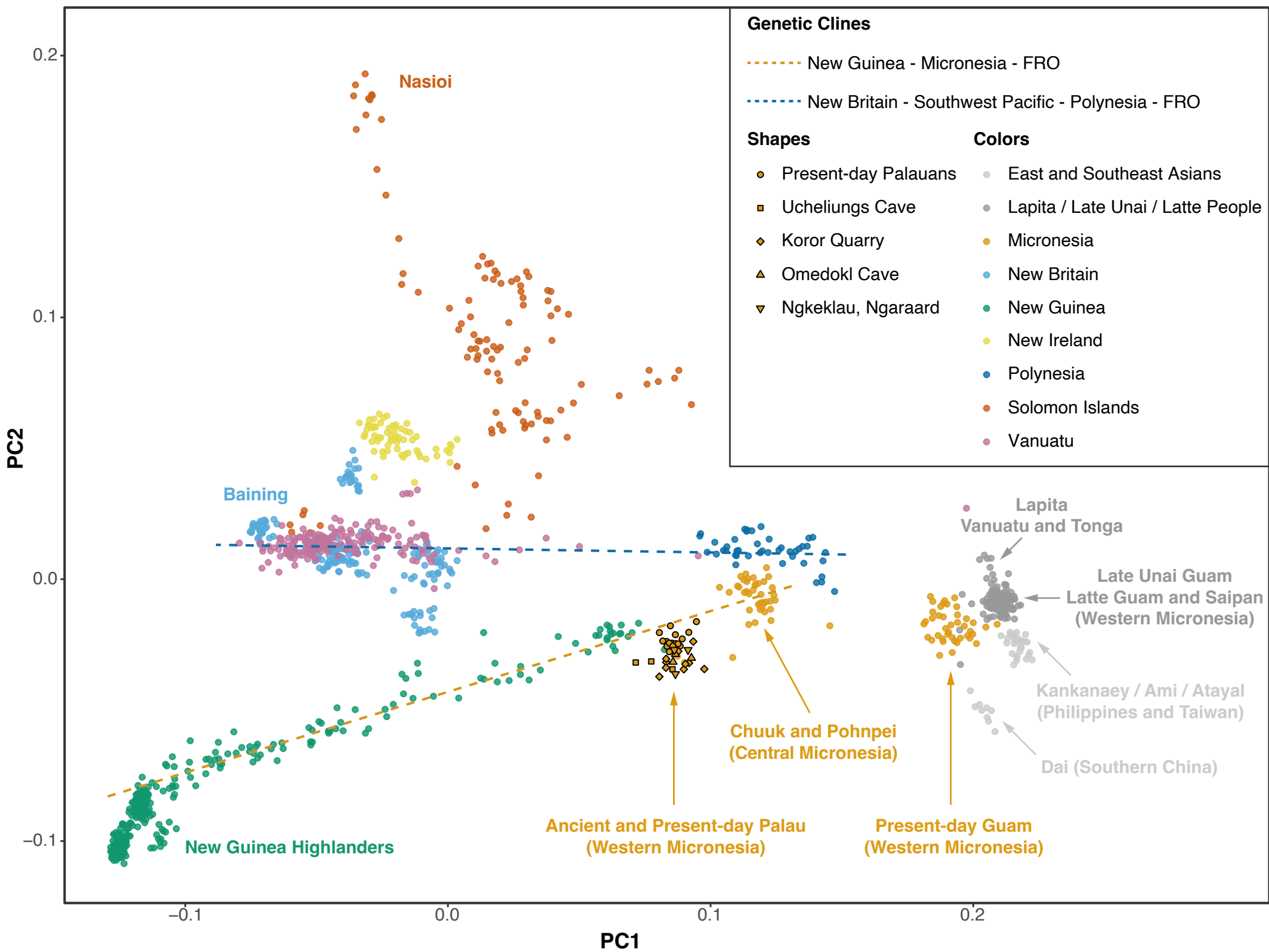

Supplement: FigureS2_PCA — Figure S2. Principal components analysis (PCA), related to Figure 1. We performed PCA on a unified set of approximately three million SNPs by integrating diverse Pacific populations genotyped on different SNP arrays (Method Details). We computed axes based on whole-genome sequencing data from three present-day populations: Papuans from the Eastern Highlands and the middle Sepik region of Papua New Guinea, Nasioi speakers from Bougainville Island in the Solomons archipelago, and Dai people from Southern China. We projected all the prehistoric and present-day individuals. For the full population list, see6. [file NIHMS2150805-supplement-FigureS2_PCA.pdf]

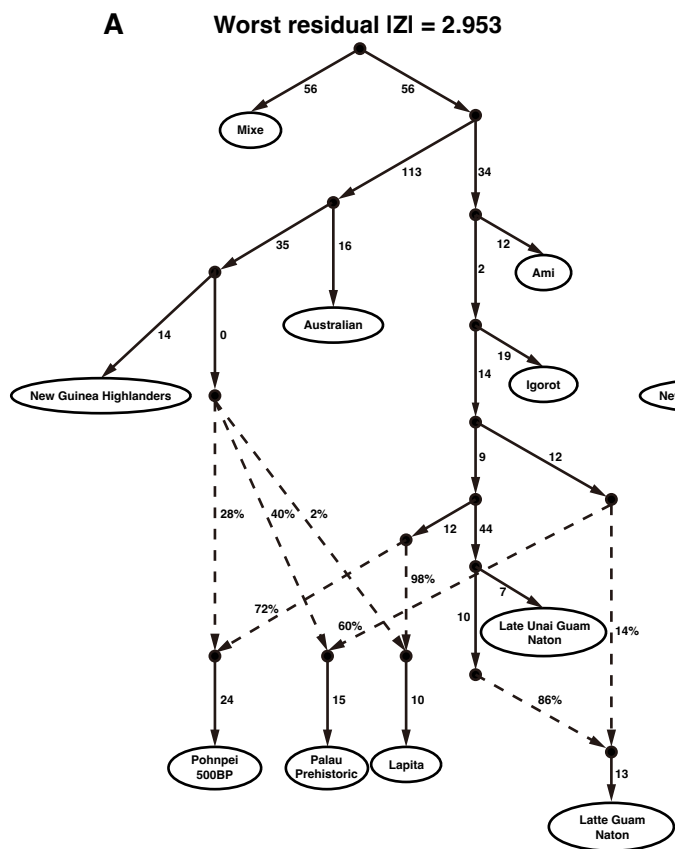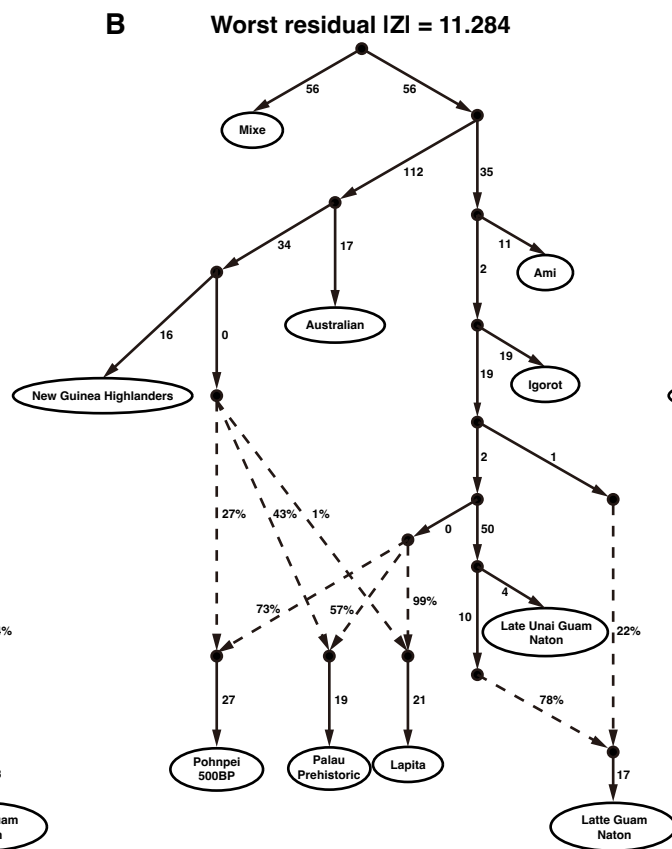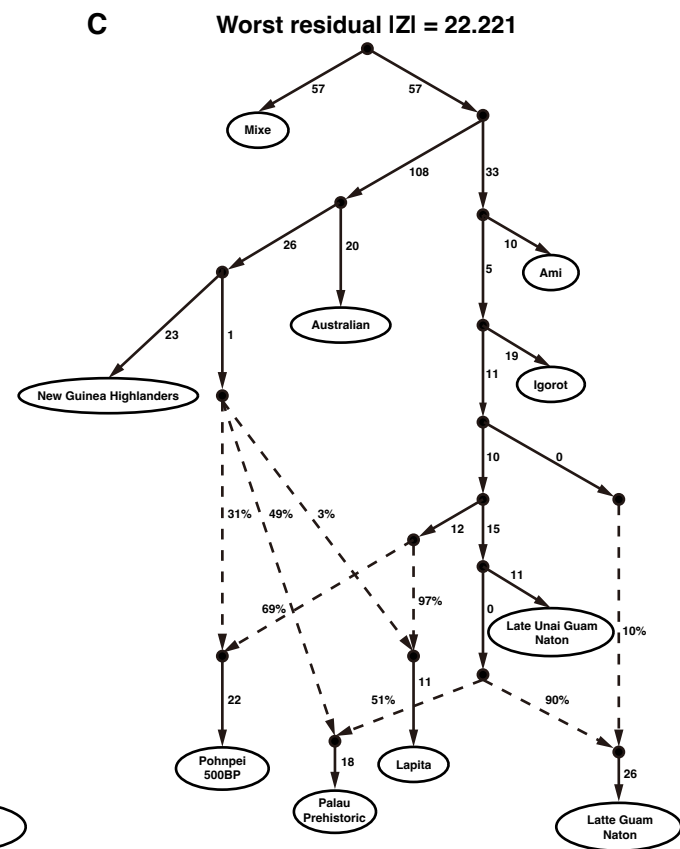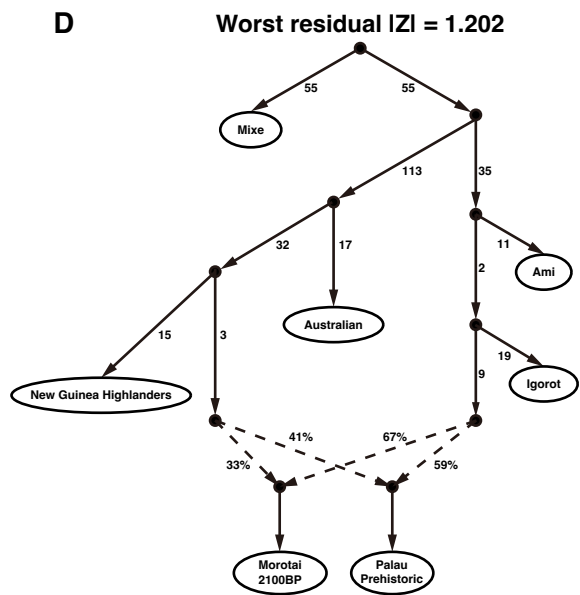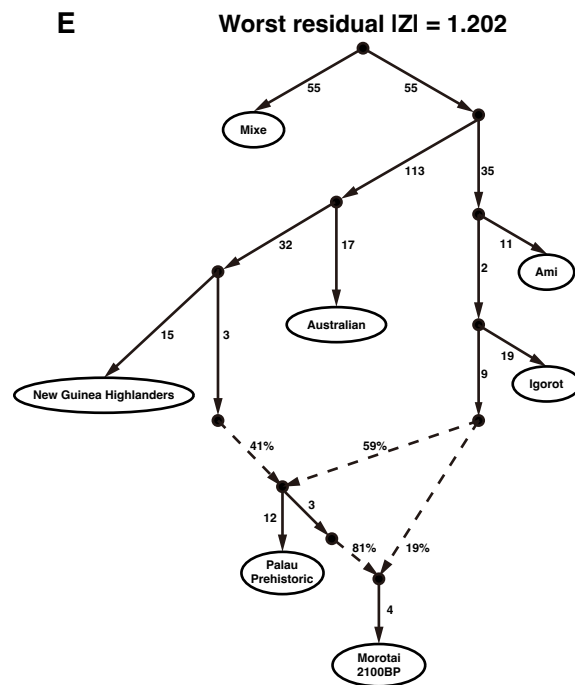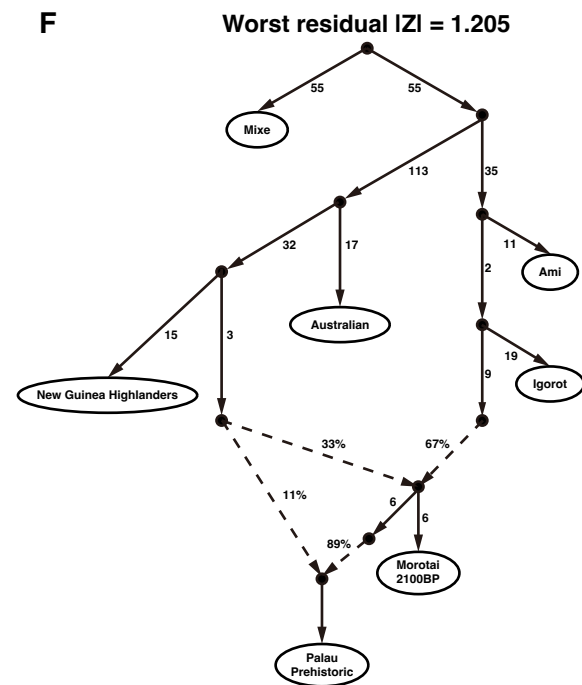

Supplement: FigureS3_Admixture_Graph — Figure S3. Fitting ancient Palau using qpGraph, related to Figures 1 and 2. A) Consistent with the previous model fitted to present-day Palauans, ancient Palauans can be fit as a mixture of ancestry related to New Guinea Highlanders and the East Asian lineage (FROPalau) that contributed to the Latte individuals in the Marianas. The maximum residual is |Z| = 2.953, which is consistent with being a good fit after correcting for the number of hypotheses tested. B) We tried modeling ancient Palauans as a mixture of ancestry related to New Guinea Highlanders and the FROSouthwestPacific lineage. The maximum residual |Z| = 11.284 for this graph, demonstrating poor fit. C) We tried modeling ancient Palauans as a mixture of ancestry related to New Guinea Highlanders and the FROMarianas lineage. The maximum residual |Z| = 22.221 for this graph, demonstrating poor fit. D) to F) Different admixture graph fits show a genetic link between ancient Palau and Northeastern Indonesia, with the maximum residual |Z| = 1.202, 1.202, and 1.205, respectively. All the models are constructed based on the autosomal intersection between the 1240K and the Twist reagents after removing sites in CpG dinucleotides (i.e. ~826,000 SNPs). Branch lengths are shown in units of average squared allele frequency divergence (multiplied by 1000, rounded to the nearest integer). [file NIHMS2150805-supplement-FigureS3_Admixture_Graph.pdf]

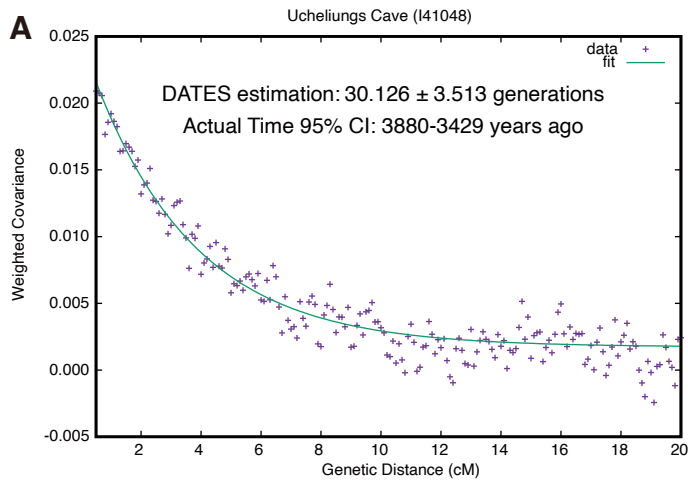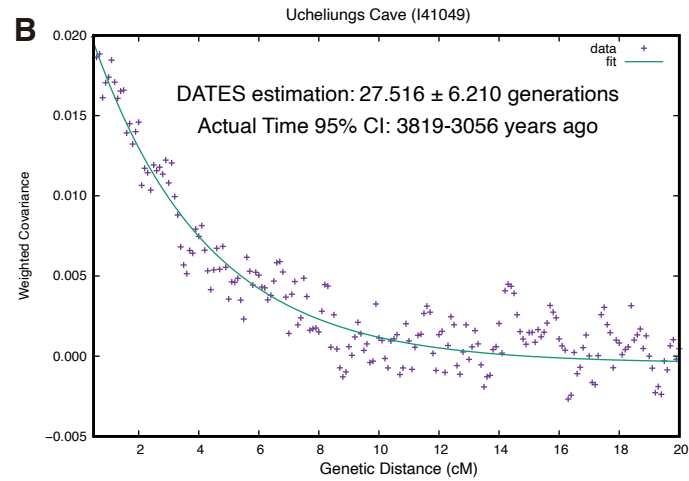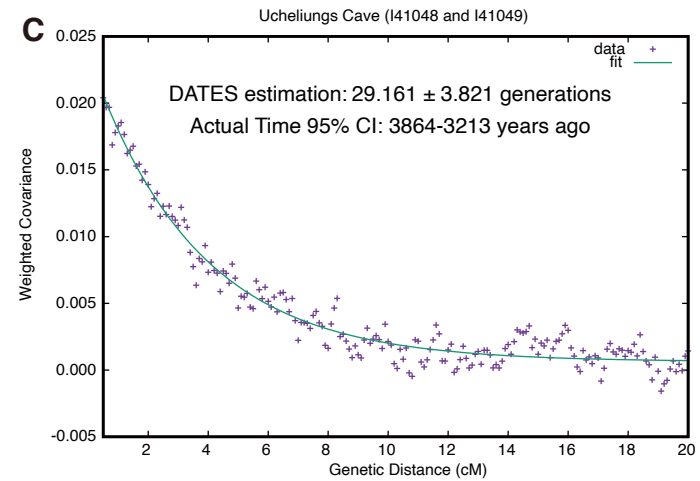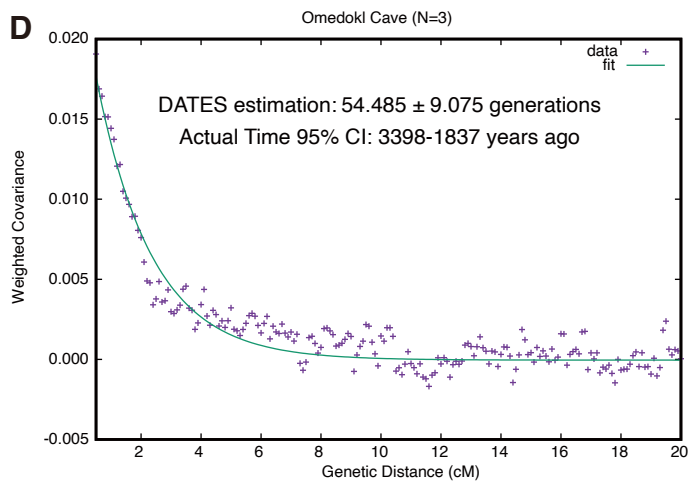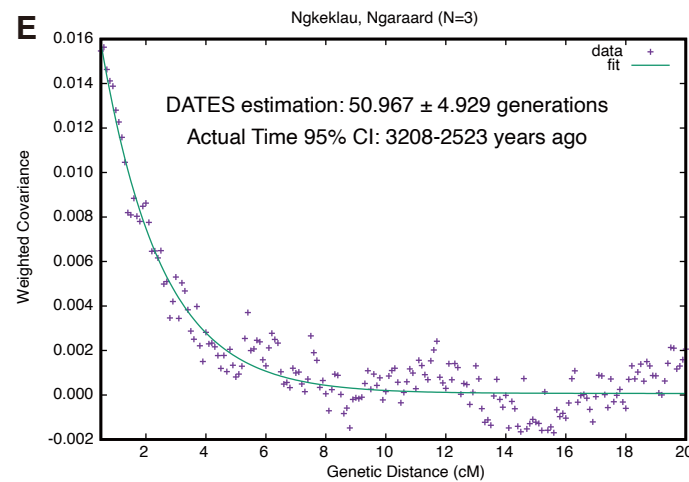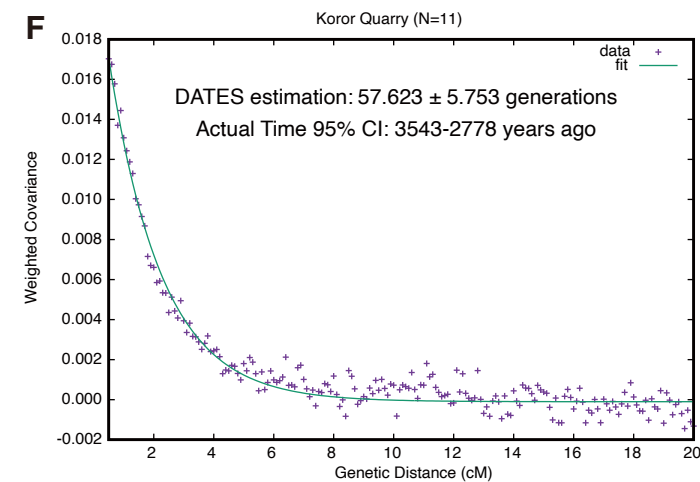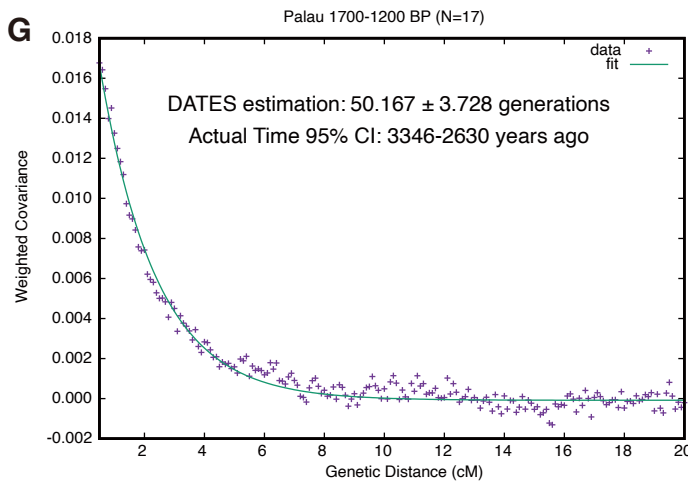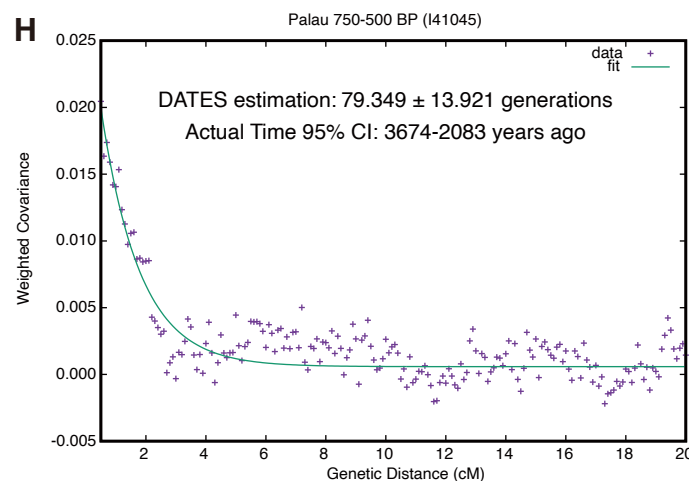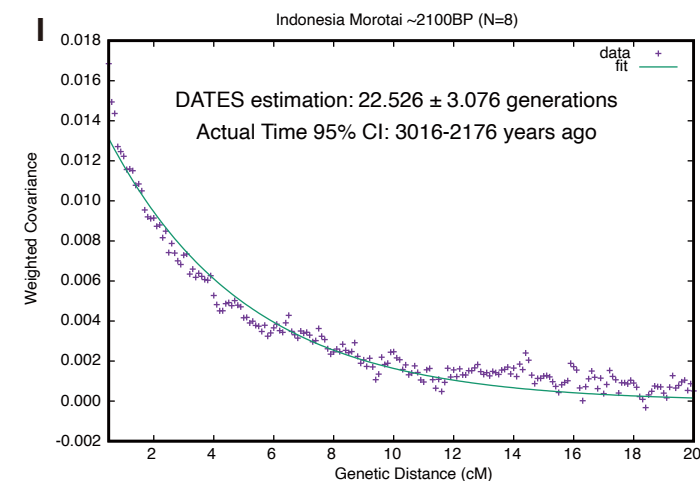

Supplement: FigureS4_Admixture_Dates_LD — Figure S4. Linkage disequilibrium (LD) for admixture date estimation, related to Figure 3. Two-way models for DATES using 56 East Asians and 25 Papuans as proxies to estimate the allele frequencies of the admixing populations. A) LD curve for Ucheliungs individual I41048; B) LD curve for Ucheliungs individual I41049; C) LD curve for the two oldest individuals from Ucheliungs Cave (2900–2500 BP) (N=2); D) LD curve for Omedokl Cave (N=3); E) LD curve for Ngkeklau (N=3); F) LD curve for Koror Quarry (N=11); G) LD curve for 17 individuals who cluster around the same date (1700–1200 BP), including two Omedokl individuals, three Ngkeklau individuals, one Ucheliungs individual, and eleven Koror individuals; H) LD curve for Omedokl individual I41045 (700–500 BP); I) LD curve for ancient Morotai, Indonesia (N=8). [file NIHMS2150805-supplement-FigureS4_Admixture_Dates_LD.pdf]
